# The multifaceted roles of NLRP3-modulating proteins in virus infection

**DOI:** 10.3389/fimmu.2022.987453

**Published:** 2022-08-30

**Authors:** James Harris, Natalie A. Borg

**Affiliations:** ^1^ Cell Biology Assays Team, Biomedical Manufacturing, Commonwealth Scientific and Industrial Research Organisation (CSIRO), Clayton, VIC, Australia; ^2^ Centre for Inflammatory diseases, Department of Medicine, School of Clinical Sciences at Monash Health, Monash University, Clayton, VIC, Australia; ^3^ Immunity and Immune Evasion Laboratory, Chronic Infectious and Inflammatory Diseases Research, School of Health and Biomedical Sciences, RMIT University, Bundoora, VIC, Australia; ^4^ Infection and Immunity Program, Monash Biomedicine Discovery Institute and Department of Biochemistry and Molecular Biology, Monash University, Clayton, VIC, Australia

**Keywords:** NLRP3, inflammasome, influenza, COVID-19, DDX3X, MIF, vimentin

## Abstract

The innate immune response to viruses is critical for the correct establishment of protective adaptive immunity. Amongst the many pathways involved, the NLRP3 [nucleotide-binding oligomerisation domain (NOD)-like receptor protein 3 (NLRP3)] inflammasome has received considerable attention, particularly in the context of immunity and pathogenesis during infection with influenza A (IAV) and SARS-CoV-2, the causative agent of COVID-19. Activation of the NLRP3 inflammasome results in the secretion of the proinflammatory cytokines IL-1β and IL-18, commonly coupled with pyroptotic cell death. While this mechanism is protective and key to host defense, aberrant NLRP3 inflammasome activation causes a hyperinflammatory response and excessive release of cytokines, both locally and systemically. Here, we discuss key molecules in the NLRP3 pathway that have also been shown to have significant roles in innate and adaptive immunity to viruses, including DEAD box helicase X-linked (DDX3X), vimentin and macrophage migration inhibitory factor (MIF). We also discuss the clinical opportunities to suppress NLRP3-mediated inflammation and reduce disease severity.

## Entry of SARS-CoV-2 and influenza virus into the host cell

SARS-CoV-2 and influenza A virus (IAV) infections have caused two major pandemics in the past century, resulting in millions of deaths. SARS-CoV-2 is an enveloped virus with a single stranded positive sense RNA genome of approximately 30,000 nucleotides. The SARS-CoV-2 genome encodes 4 structural proteins (spike, envelope, membrane, nucleocapsid), 16 non-structural proteins (nsp1-16) and multiple accessory proteins that together have proven masterful at entering host cells and manipulating host machinery to replicate their genome. The SARS-CoV-2 spike protein studs the viral envelope and facilitates entry into host cells with the help of several host cellular proteases, including furin and type II transmembrane serine protease (TMPRSS2) (1–3). These host proteases cleave the spike protein into its two subunits; the S1 domain, which interacts with the angiotensin converting enzyme 2 (ACE2) receptor on the host cell surface, and the S2 domain which mediates virus-host cell membrane fusion (4). Besides ACE2, SARS-CoV-2 engages with alternate entry receptors including heparan sulfate (5), cluster of differentiation 147 (CD-147) (6), neuropilin-1 (7) and the C-type lectin receptors DC-SIGN (8, 9), L-SIGN (10) and sialic acid binding Ig like lectin 1 (SIGLEC1) (11). Together, these enable the release of SARS-CoV-2 genomic RNA into the host cell cytoplasm and/or induction of the innate immune response. IAV, on the other hand, is a negative stranded RNA virus with a genome approximately half that of SARS-CoV-2 that encodes 10 essential proteins, including matrix proteins 1 (M1) and 2 (M2), three RNA polymerases, nucleoprotein (NP), non-structural proteins 1 (NS1) and 2 (NS2) and the surface antigens haemagglutinin (HA) and neuraminidase (NA) (12). The HA protein mediates viral entry by preferentially binding to α2,6-linked sialic acid receptors, typically on the surface of host epithelial cells, and enters the cell by receptor-mediated endocytosis (13). Upon entry of IAV and SARS-CoV-2, the host translational machinery is hijacked to replicate viral RNA and transcribe viral proteins required for the release of mature virions from the host cell and to subvert immune defenses. Thus, the clinical outcome of viral infection is a battle between viral pathogenesis and the host immune response to infection.

## Innate immune response to viral infection

**Figure 1:**
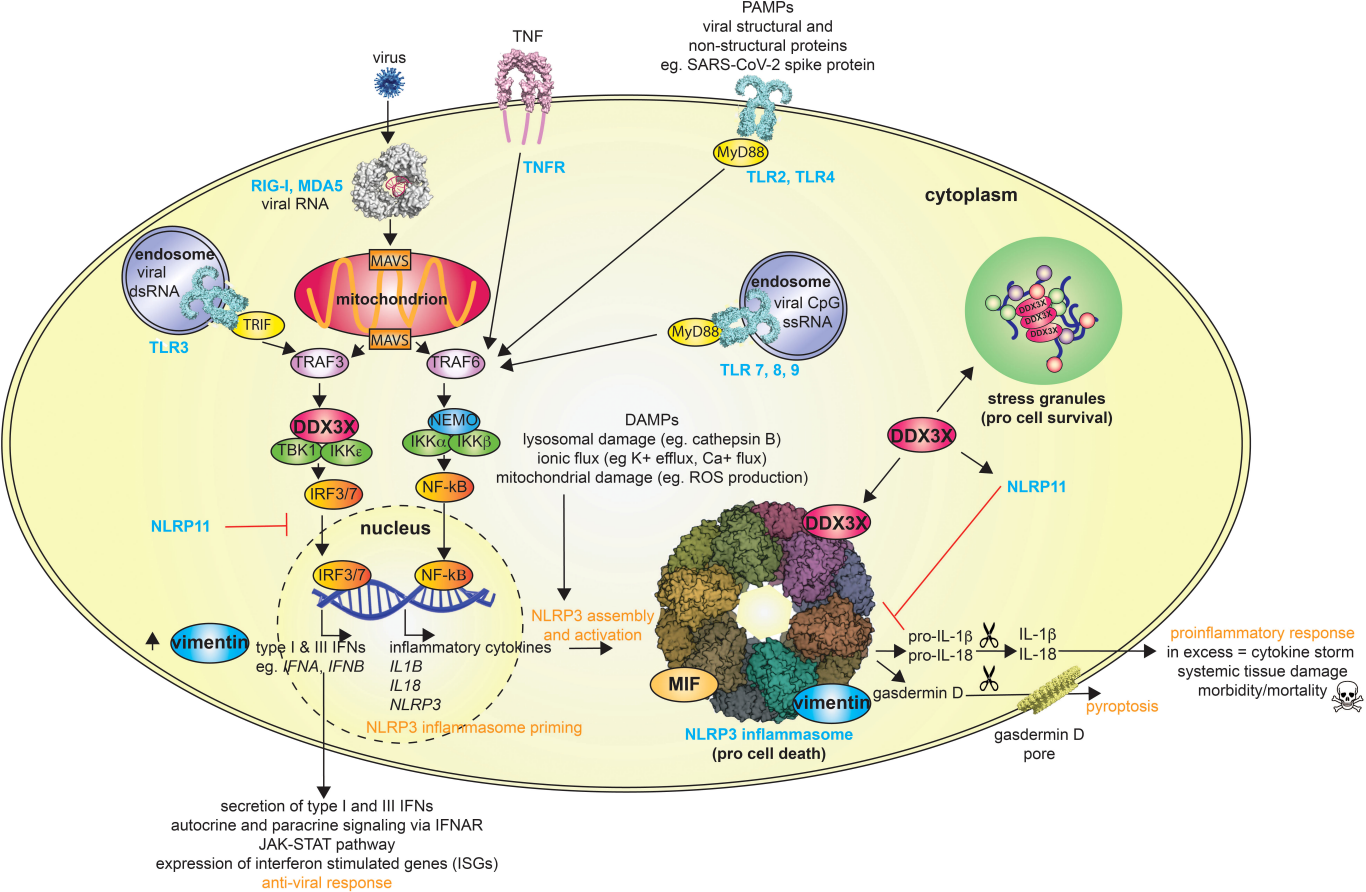
Overview of host defense following viral infection and multiple roles of DDX3X, vimentin and MIF. The scissors symbol represents enzymatic cleavage, and the upward arrow symbol indicates increased protein levels. RIG-I, retinoic acid-inducible gene I; MDA5, melanoma differentiation-associated 5; TNF, tumor necrosis factor; TNFR, TNF receptor; TRAF, TNF receptor associated factor; MAVS, mitochondrial antiviral signaling protein; TRIF, TIR domain–containing adapter-inducing interferon-β; TBK1, TANK-binding kinase 1; DDX3X, DEAD-box protein 3X; IKK, I-kappa-B kinase; IRF, interferon regulatory factor; NF-κB, nuclear factor-κB; NEMO, NF-κB essential modulator; TLR, toll-like receptor; MyD88, myeloid differentiation primary response 88; MIF, macrophage migration inhibitory factor; NLRP, nucleotide-binding oligomerization domain (NOD)-like receptor; IL, interleukin; ROS, reactive oxygen species.

The innate immune system detects and responds to infectious agents to provide rapid frontline defense. This frontline defense is achieved using immune sentinels, or pattern recognition receptors (PRRs), that recognize exogenous pattern-associated molecular patterns (PAMPs) and endogenous damage-associated molecular patterns (DAMPs). In the case of viral infection, PAMPs may be associated with the viral particle, such as the SARS-CoV-2 spike protein which induces inflammation *via* the C-type lectin receptors (14) or toll-like receptor (TLR) 2 (15), or intermediates of viral replication, such as SARS-CoV-2 double-stranded RNA which activates the retinoic acid-inducible gene I (RIG-I)-like receptors (RLRs) or TLRs (16, 17) (**Figure 1**). Virus-induced cell lysis can also lead to the release of host products or DAMPs, that similarly initiate innate immune responses. As a result, viral infection can trigger multiple signaling cascades leading to the release of multiple cytokines, including the IL-1 family cytokines IL-1β and IL-18, and type I and type III interferons (IFNs) (**Figure 1**). Type I IFNs, such as IFN-α and IFN-β, signal through the type I IFN receptor (IFNAR) complex, whereas type III IFNs, termed IFN-Λs, signal *via* the IFN lambda receptor 1 (IFNLR1) and interleukin-10 receptor 2 (IL-10R2) complex (18). The engagement of IFNs with their cognate receptors activates the receptor-associated protein tyrosine kinases Janus kinase 1 (JAK1) and tyrosine kinase 2 (TYK2), which phosphorylate signal transducer and activator of transcription 1 (STAT1) and STAT2 (19). The dimerization and nuclear translocation of STAT1 and STAT2 leads to the activation of hundreds of interferon-stimulated genes (ISGs) to restrict viral replication and limit viral spread (**Figure 1**). IL-1 family cytokines are potent pro-inflammatory and pyrogenic immunomodulators whose release in response to virus replication activates multiple immune genes and recruits immune cells to the site of infection. The processing and release of both IL-1β and IL-18 is typically dependent on the activation of an inflammasome, in the case of viruses most commonly the absent in melanoma 2 (AIM2) and/or nucleotide-binding oligomerization domain (NOD)-like receptor protein 3 (NLRP3) inflammazome (20).

## The NLRP3 inflammasome

NLRP3 is a cytoplasmic PRR that is mainly expressed in monocytes and macrophages. It consists of an N-terminal pyrin domain followed by a central NACHT [NAIP (neuronal apoptosis inhibitor protein), CIITA (MHC class II transcription activator), HET-E (heterokaryon incompatibility), TP1 (telomerase-associated protein 1)] domain and C-terminal leucine-rich-repeats (LRRs) (21). NLRP3 is normally maintained in an autoinhibited state (22), however, it is activated upon sensing diverse stress signals and signatures of infection, leading to the assembly of the NLRP3 inflammasome, which can elicit both inflammatory and antiviral responses. Canonical activation of the NLRP3 inflammasome generally requires a priming and then activation step (23), although NLRP3 inflammasomes may also be formed in the absence of priming (24). The priming signal, which can be initiated by various PAMPs and DAMPs, activates nuclear factor-κB (NF-κB), which upregulates the expression of pro-IL-1β, pro-IL-18 and NLRP3, thus priming NLRP3 for inflammasome formation. The second activating signal again stems from diverse inducers/indicators of stress (e.g., pore forming toxins such as nigericin), however, all have been reported to induce potassium (K^+^) efflux, suggesting that this is a unifying process required for inflammasome assembly (25). The activating signal elicits a change in NLRP3 conformation that allows it to oligomerize and recruit ASC (apoptosis-associated speck-like protein containing a CARD) *via* a pyrin-pyrin domain interaction. ASC in turn, recruits pro-caspase 1 *via* a CARD-CARD interaction, together forming an assembled NLRP3 inflammasome (**Figure 1**). Within this assembled inflammasome, pro-caspase-1 undergoes autocatalytic activation and cleaves pro-IL-1β, pro-IL-18 and the pore forming protein gasdermin D, which prompts gasdermin D to oligomerize and form pores in the cell membrane (26–29). These pores allow release of the mature and active pro-inflammatory cytokines IL-1β and IL-18, which is commonly coupled with cell death *via* pyroptosis (30) (**Figure 1**). This process destroys infected cells, which in the case of viral infection prevents them from fueling further replication, and releases the cell contents, allowing further amplification of the innate immune response.

Multiple RNA viruses, and some DNA viruses, are sensed by, and activate, the NLRP3 inflammasome but precisely how is not completely understood. Priming is most likely through activation of TLR3 and/or TLR7, which recognize nucleic acids. Activation of NLRP3, on the other hand, may occur through multiple mechanisms. Some viruses, including vesicular stomatitis virus (VSV) and encephalomyocarditis virus (EMCV) may activate NLRP3 by increasing K^+^ efflux as a result of lytic cell death (31). Alternatively, NLRP3 may recognize, directly or indirectly, viral nucleic acids (32) and/or proteins, including the IAV M2 ion channel and/or PB1-F2 peptide aggregates (33, 34) and SARS-CoV-2 nucleocapsid protein, nsp6 and spike protein (35, 36). Both IAV and SARS-CoV-2 are also able to suppress NLRP3 inflammasome activation *via* their non-structural protein 1 (NS1) and envelope proteins, respectively. Interestingly, the IAV protein PB1-F2, while an activator as an aggregate, can also inhibit NLRP3 following translocation into the mitochondria (37, 38).

## New players in NLRP3 inflammasome activation

Although it is well-established that the NLRP3 inflammasome is a multi-protein complex that requires assembly for its activation, the events leading up to, and regulating, NLRP3 inflammasome activation are unclear. Identifying new players that regulate NLRP3 inflammasome activation furthers our understanding of this critical process and reveals new ways to block aberrant activation which occurs in several disease states, including severe SARS-CoV-2 and IAV infection. While new proteins undoubtedly remain to be discovered, recent studies have highlighted critical roles for three proteins in NLRP3 inflammasome activation and assembly: DEAD-box helicase 3X (DDX3X), vimentin and macrophage migration inhibitory factor (MIF).

### DEAD-box helicase 3X

The family of DEAD box helicase proteins, including DDX3X, are comprised of ATP-dependent RNA helicases that unwind RNA or remodel protein-RNA complexes. As a result, they are involved in almost all stages of RNA metabolism in eukaryotic cells, including transcription, splicing, nuclear export and mRNA translation. DDX3X, which is encoded on the X-chromosome, has a role to play in multiple biological functions including innate immunity and the cellular stress response. We and others have previously shown that DDX3X plays a prominent role in enhancing the RLR-mediated type I interferon (IFN) antiviral response by associating with various RLR signaling molecules including TBK1 (TANK-binding kinase I) and IKKε (I-kappa-B kinase-ε) and direct interaction with the *IFNB1* promoter (39–43) (**Figure 1**). DDX3X also participates in the formation of cytoplasmic stress granules, membraneless cytosolic bodies that form during the cell stress response, as well as during viral infection (44, 45). Stress granules are highly dynamic structures that form when translation initiation is stalled and they contain a diverse proteome of RNA-binding and other proteins, along with untranslated mRNAs (46, 47). The biological functions of stress granules are not well understood, but by sequestering specific cytosolic constituents, they modulate multiple signaling pathways, including mechanistic target of rapamycin (mTOR), receptor for activated C kinase 1 (RACK1) and tumor necrosis factor (TNF) receptor-associated factor 2 (TRAF2) (47). DDX3X dysfunction is associated with a wide range of diseases, including cancer, inflammation, female intellectual disability and viral infections (48–50).

DDX3X has a homologue encoded on the Y-chromosome known as DDX3Y. With 92% sequence identity, DDX3X and DDX3Y have functionally redundant roles in translation (51). Despite this, Szappanos et al. recently showed that male and female mice lacking DDX3X in the hematopoietic system differ in their susceptibility to the intracellular bacterial pathogen, *Listeria monocytogenes* (52). This is attributed to the distinct expression profiles of DDX3X versus DDX3Y, with immune cells exhibiting differential sex biased *DDX3X/DDX3Y* gene expression (52, 53).

#### DDX3X and NLRP3

DDX3X has been attributed a role in NLRP3 inflammasome activation as cultured cell lines deficient in *Ddx3x* (54, 55) and bone marrow derived macrophages (BMDMs) that lack DDX3X demonstrated impaired release of IL-1β and IL-18 (55). Based on co-immunoprecipitation analysis, it is proposed that DDX3X binds the central NACHT domain of NLRP3 to facilitate NLRP3 oligomerization and inflammasome assembly, both of which are key to NLRP3 inflammasome activation. However, a direct interaction between NLRP3 and DDX3X has not yet been confirmed using recombinant proteins.

Intriguingly, two independent studies have linked stress granule assembly with inhibition of NLRP3 inflammasome activation in response to various cellular stressors, including viral infection (55, 56). Further, BMDMs derived from mice that lacked DDX3X in the myeloid compartment were used to establish DDX3X as a pivotal link between these two mutually exclusive cellular responses to stress. Notably, when DDX3X is incorporated into stress granules it is no longer available to partake in NLRP3 inflammasome activation, thus favoring cell survival (**Figure 1**) (55). Therefore, DDX3X availability influences cell-fate decisions in stressed cells.

DDX3X also binds NLRP11, another member of the NOD-like receptor family, but unlike NLRP3 NLRP11 does not form a classical inflammasome and early studies on its function suggest roles in regulating NF-κB and type I IFN (57–59). Further, and as per stress granule formation, NLRP11 has been shown to inhibit NLRP3 inflammasome activation, a phenomenon again attributed to DDX3X sequestration, this time by NLRP11. Perplexingly, NLRP11 was later identified as an essential component required for NLRP3 inflammasome activation (60). How NLRP11 differentiates between its inhibitory and activating effects on NLRP3 inflammasome activation is currently unclear, however it is possible that DDX3X influences this process. Despite the conserved architecture of NLRP3 and NLRP11, DDX3X binds the LRR domain of NLRP11 as opposed to the NACHT domain of NLRP3. DDX3X might therefore concomitantly bind both NLRP3 and NLRP11, a concept that should be further investigated.

#### DDX3X and immunity to viruses

DDX3X plays multiple roles in host antiviral defense following the detection of viral RNA, and binds to, or is bound by, numerous viral proteins from diverse virus families, the outcomes of which determine whether DDX3X plays a pro- or antiviral role in infection. DDX3X plays antiviral roles in vaccinia virus and IAV infection. As such, vaccinia virus and IAV encode proteins to counteract DDX3X. The K7 protein binds to DDX3X to block IFN-β production (61) (**Table 1**). During IAV infection DDX3X regulates stress granule formation, the type I IFN response and NLRP3 inflammasome activation (**Table 1**) (56, 71). On the other hand, the IAV NS1 protein, which interacts with DDX3X in an RNA-independent manner, inhibits both stress-granule formation and the type I IFN response, however whether NS1 promotes or inhibits NLRP3 inflammasome activation is contentious (39, 56, 71–75). Despite the influence of NS1, DDX3X protects IAV-infected mice against severe lung pathology and viral spread, thus aiding their survival (**Table 1**) (56).

**Table 1:** DDX3X, vimentin and MIF are host proteins with roles in antiviral activity and NLRP3 activation.

NLRP3 inflammasome mediator	Virus	Role in infection	References
**DDX3X**			
	Human immunodeficiency virus type I	DDX3X is required for nuclear export of incompletely spliced human immunodeficiency virus type I RNA transcripts. DDX3X inhibitors suppress human immunodeficiency virus type I replication.	(62, 63) (64–68)
	SARS-CoV-2	DDX3X catalytic inhibitor RK-33 suppresses viral load in Calu-3 cells. DDX3X interacts with SARS-CoV-2 nucleoprotein in infected Vero cells and may subvert stress granule machinery.	(69) (70)
	Influenza A virus	DDX3X plays a role in NLRP3 inflammasome activation, stress granule assembly and production of type I interferon; stress granule assembly and type I interferon production is blocked by influenza virus NS1 protein, whereas its effect on NLRP3 is contentious.	(39, 56, 71–75)
	Respiratory syncytial virus	DDX3X binds to respiratory syncytial virus M2 mRNA to initiate translation which enables the onset of genome replication. DDX3X catalytic inhibitor RK-33 suppresses viral load in Vero cells.	(76) (77)
	Dengue virus	DDX3X binds dengue virus capsid and NS5 proteins, but knockdown showed differential effects on virus production. DDX3X inhibitors suppress dengue virus infection in Huh-7 and Vero cells. DDX3X is required for IFN-β release and inhibits virus replication in HEK-293 cells.	(78, 79) (77, 80, 81) (82)
	Vaccinia virus	Vaccinia virus K7 protein forms a complex with DDX3X and inhibits IFN-β.	(61)
	Zika virus	DDX3X directly binds and unwinds *in vitro* transcribed Zika virus 5’ terminal regions. DDX3X catalytic inhibitor RK-33 suppresses viral load in Vero cells.	(83) (77)
	West Nile virus	DDX3X catalytic inhibitors have antiviral effects after the virus entry process in Huh-7, Vero and A549 cells. DDX3X inhibitors suppresses viral load in Vero cells.	(84) (77, 80, 85)
	Human parainfluenza virus type 3	DDX3X catalytic inhibitor RK-33 suppresses viral load in Vero cells.	(77)
	Hepatitis B virus	DDX3X binds hepatitis B virus polymerase and inhibits hepatitis B virus reverse transcription following nucleocapsid assembly. DDX3X knockdown or overexpression increased or decreased hepatitis B RNA, respectively.	(87) (88)
	Hepatitis C virus	DDX3X interacts with hepatitis C virus 3’ untranslated regions and facilitates viral assembly/infection. DDX3X interacts with hepatitis C virus core protein, but the importance for replication is contentious.	(89–92) (89–91, 93–97)
	Japanese encephalitis virus	Knockdown of DDX3X inhibits Japanese encephalitis virus replication.	(86)
		DDX3X binds and unwinds Japanese encephalitis virus 3’ and/or 5’ untranslated regions and positively regulates viral protein expression.	(83, 86)
**Vimentin**			
	Human immunodeficiency virus type I	HIV-1 infectivity reduced in MT4 cells stably transfected with shRNA targeting vimentin.	(98)
	SARS-CoV/SARS-CoV-2	Vimentin interacts with the SARS-CoV spike protein, possible role in virus entry in Vero cells. Vimentin interacts with SARS-CoV-2 spike protein and facilitates virus entry *via* ACE2 in HEK-293 cells. Vimentin, ACE2 and SARS-CoV-2 co-localize in structures consistent with primary cilia in multiple cell types. Vimentin-targeting compound ALD-R491 reduced ACE2-mediated infection of lentivirus pseudotyped with SARS-CoV-2 spike protein in HEK-293 cells, increased anti-microbial activity of macrophages, activates T regulatory cells and had therapeutic efficacy in a mouse adapted model of SARS-CoV-2 infection.	(99) (100) (101) (102)
	Influenza A virus	Influenza A infection is reduced in *Vim* ^-/-^ mouse embryonic fibroblasts. Vimentin affects the distribution of endosomes and their acidification during influenza A virus infection impairing viral genome release from endosomes. Vimentin interacts with the PB2 subunit of the influenza A virus polymerase to enhance its activity and virus replication.	(103) (103) (104)
	Dengue virus	Viral load is increased in *Vim* ^-/-^ human brain microvascular endothelial cells and *Vim* ^-/-^ SV129 suckling mice infected with Dengue virus had increased viral load and more severe brain damage. Vimentin interacts with NS4A in Huh-7 cells to facilitate viral replication.	(105) (106)
	Vaccinia virus	Intracellular vimentin is found around viral factories in HeLa cells and within viroplasm foci, where virus assembly occurs.	(107)
	Cytomegalovirus	Loss/disruption of vimentin in cells reduced viral entry and slowed intracellular capsid transport.	(108)
	Human Enterovirus	Vimentin regulates the synthesis of human enterovirus non-structural proteins 2A, 3C, and 3D which regulate host cell processes, including prolonged cell survival.	(109)
	Human papillomavirus	Cell surface expressed vimentin restricts binding to cell surface receptor and internalization into pgsD677, HaCaT, HeLa, and NIKS cells.	(110)
	Hepatitis C virus	Vimentin binds the hepatitis C core protein and influences its proteasomal-mediated degradation to impact virus replication in Huh-7 cells.	(111)
	Foot and mouth disease virus	Intracellular vimentin interacts with virus non-structural Protein 3A (NS3A) in foetal bovine kidney cells and hinder viral replication in PK-15 cells.	(112)
**MIF**			
	Human immunodeficiency virus type I	Plasma MIF is increased in patients with human immunodeficiency virus type I and is released in greater amounts by infected PBMC. MIF increases viral replication of CCR5- and CXCR4-trophic isolates, while anti-MIF antibody inhibits human immunodeficiency virus type I replication in PBMC.	(113)
	SARS-CoV-2	MIF is increased in patients with mild and severe SARS-CoV-2 and may be associated with impaired organ function and increased pulmonary arterial hypertension.	(114–116)
	Influenza A virus	MIF is involved in influenza virus replication, contributes to an overactive inflammatory cytokine response and aggravated disease. Antisense oligodeoxyribonucleotide targeting MIF inhibits propagation of influenza virus (H1N1) in A549 cells. MIF interacts with influenza virus protein PB1-F2 protein. MIF is released from necrotic lung epithelial cells infected with influenza virus.	(117) (118) (119) (120)
	Respiratory syncytial virus	Respiratory syncytial virus infection increases *Mif* MRNA expression in mouse macrophages and inhibition of MIF with ISO-1 (small molecule inhibitor) inhibits RSV-induced release of TNF, MCP-1 and IL-10.	(121)
	Dengue virus	MIF inhibits migration of dengue virus-infected macrophages to limit virus spread but is also involved in pathogenesis of dengue virus infection, including an overactive inflammatory cytokine response, viral replication, vascular permeability and leakage.	(122–128)
	Zika virus	MIF inhibits migration of Zika virus-infected macrophages to limit virus spread; Zika virus blocks NF-κB signaling to reduce MIF expression and its role in inhibiting macrophage migration.	(129)
	West Nile virus	MIF facilitates West Nile virus neuroinvasion and replication and causes an overactive inflammatory response. High expression MIF polymorphisms associated with West Nile virus neuroinvasion/encephalitis in North American patients.	(130–132)

Although many viruses inhibit stress granules, several viruses interact with stress granule components to fulfil their replication cycle. Hepatitis C virus (HCV) infection induces an interaction between DDX3X and the HCV 3’ untranslated region which prompts DDX3X and IKKα to associate with stress granules (89, 90). These in turn bind to HCV protein and RNA elements to form the HCV replication complex (**Table 1**). Intriguingly, siRNA knockdown of DDX3X or key stress granule proteins revealed a beneficial role for DDX3X and stress granules in HCV infection (90–92). This role may serve to prolong cell survival and favor persistent HCV infection (133). DDX3X also binds to the HCV core protein which associates with lipid droplets essential for infectious HCV production, however the role of this interaction in infection is contentious (89–91, 93–97).

In addition to HCV replication, DDX3X promotes the replication of many other viruses as exemplified by DDX3X knockdown/knockout severely limiting human immunodeficiency virus type I (HIV-1) (62, 63), hepatitis B (HBV) (88), Japanese encephalitis virus (JEV) (86) and SARS-CoV-2 (70) infectious virus production. Further, we and others have also shown that inhibitors that suppress DDX3X activity *in vitro* can limit infectious virus production by HCV (80), respiratory syncytial virus (RSV) (76, 77), West Nile virus (WNV) (77, 80, 85), Zika virus (77), SARS-CoV-2 (70), HIV-1 (64–67) and drug-resistant HIV-1 (68). Therefore, for these many viruses, DDX3X plays a pro-viral role in replication. On the other hand, it is worth noting that both pro- and anti-viral roles have been attributed to DDX3X in dengue virus (DENV) infection. While DDX3X inhibitors and knockdown suppress the replication of DENV or a DENV replicon (77, 78, 80), Kumar and colleagues (79) showed that DDX3X knockdown led to an increase in DENV viral titer and conversely DDX3X overexpression inhibited DENV replication. Likewise, DDX3X was shown to suppress DENV infection by activating IFN (82). Thus, DDX3X may have contrasting roles to play in the biology of the infected cell, with differing implications for the host response to DENV.

DDX3X appears the subject of a complex ‘tug of war’, where its involvement in stress granule formation, or binding to NLRP11, prevents its role in NLRP3 inflammasome activation. These findings highlight that DDX3X plays a pivotal role in the ‘cross-talk’ been various arms of innate immune defense and therefore its role might be expected to alter during the course of a viral infection. Further influencing DDX3Xs role in innate immune defense is the host/virus interplay, which is particularly pertinent given the vast number of viruses that block or hijack DDX3X function during infection (**Table 1**). It is tempting to speculate that multiple viruses that hijack DDX3X not only utilize its helicase activity to service their replication, but in binding to DDX3X may concomitantly influence cell fate decisions, evidence for which is now mounting (56, 90). Thus, rather than capturing snapshots of DDX3X’s function during viral invasion, it should instead be systematically explored at multiple time points during viral infection.

### Vimentin

Vimentin is a type III intermediate filament cytoskeletal protein expressed mostly in mesenchymal cells, including cells of the immune system. Vimentin filaments modulate a wide variety of cellular functions, including cytoskeletal stabilization, migration, adhesion, division, organelle homeostasis, signaling, aggresome formation, lipid metabolism and gene regulation. Vimentin has important roles to play in immune responses, including inflammation, wound healing, fibrosis and the host response to infectious pathogens, including viruses (134). Phosphorylated vimentin can also be expressed at the surface of cells, including macrophages and neutrophils and is secreted in response to various cellular processes, including cell activation, apoptosis, senescence and stress (135–138).

#### Vimentin and NLRP3

Vimentin filaments have been found to play an important role in several signaling pathways, potentially acting as a protein scaffold that stabilizes signaling complexes. Moreover, vimentin has been shown to interact with signaling molecules, including phosphorylated extracellular-regulated kinase 1/2 (pERK1/2) and NOD2, which it binds *via* the LRR, a protein binding domain shared by multiple NLRs, including NLRP3 (139, 140). Based on this, dos Santos and colleagues investigated whether vimentin had a role to play in NLRP3 inflammasome activation (141). In response to intraperitoneal injection of lipopolysaccharide (LPS) or intratracheal administration of asbestos or bleomycin, measures of acute lung injury (ALI), including histopathology and levels of caspase-1 and IL-1β, were attenuated in *Vim* ^-/-^ mice, compared to wild type (WT) controls. Similarly, NLRP3 inflammasome activation, measured by caspase-1 activation and release of mature IL-1β, was significantly reduced in primary alveolar macrophages from *Vim* ^-/-^ mice treated with LPS and ATP *ex vivo*, as well as in human THP-1 macrophages transfected with siRNA against *VIM* and treated with LPS and asbestos or monosodium urate (MSU) crystals (141). Likewise, mouse immortalized BMDMs treated with the vimentin-binding anti-angiogenic compound withaferin A show significantly reduced NLRP3 activation in response to LPS and the bacterial toxin nigericin (122). Moreover, vimentin was found to co-localize and interact with NLRP3 in THP-1 cells by confocal microscopy, co-immunoprecipitation and using a bio-layer interferometer (141). Similarly, super resolution microscopy has shown NLRP3 to co-localize with to filamentous vimentin (122) (**Figure 1**). Nonetheless, whether intermediate filaments are involved in NLRP3 activation, or whether vimentin acts independently of them, is not yet clear. Vimentin is also present on particles which are converted into short motile intermediate filaments, or ‘squiggles’, the movement of which depends on microtubules (142). In this context, it is interesting that tubulin inhibitors, including colchicine, have been shown to inhibit NLRP3 activation (143, 144). Colchicine is an anti-inflammatory drug that is currently approved for treatment of acute flares of gout and familial Mediterranean fever, as well as off-label for multiple inflammatory and autoimmune conditions (145). More recently, it has been looked at as a potential treatment for SARS-CoV-2 (146).

#### Vimentin and immunity to viruses

Studies have highlighted a role for vimentin in the entry, replication and release of many different viruses (**Table 1**). Extracellular/cell surface vimentin has been implicated in the entry of SARS-CoV, SARS-CoV-2, DENV, foot-and-mouth disease virus (FMDV) and human papillomavirus 16 pseudovirions (99, 100, 110, 112, 147). Intracellular vimentin, on the other hand, has been shown to regulate the activity, transport and/or replication of IAV, DENV and HCV (103, 106, 111). Conversely, some viruses target and modify vimentin to aid their replication, including DENV, FMDV and group B human enteroviruses (106, 109, 148). In the case of IAV infection, vimentin appears to be critical for host cell responses, as levels of virus mRNA and protein, along with virus titres, were significantly reduced in infected *Vim* ^-/-^ mouse embryonic fibroblasts (103). Interestingly, these *Vim* ^-/-^ cells also demonstrated impaired endosomal distribution and acidification, leading to the accumulation of virions in late endosomes. It is proposed that this may, in turn, block the release of the viral genome (103). For SARS-CoV-2, surface vimentin appears to be critical for virus entry. Vimentin binds to the SARS-CoV-2 spike protein and facilitates the entry of pseudotyped viruses expressing the spike protein into HEK-293 and A549 cells expressing ACE2 (100). Specifically, vimentin binds to the receptor binding domain (RBD) of the spike protein, but at a different site to ACE2 (100).

In addition to cytoskeletal rearrangement and intracellular trafficking, vimentin can potentially modulate host cell immune/inflammatory responses to viruses. The role of vimentin in NLRP3 assembly and activation may underlie the observation that *Vim* ^-/-^ mice infected with a lethal dose of IAV show decreased mortality and measures of alveolar and lung capillary damage compared to WT animals (149). Viral replication and clearance were not affected by loss of vimentin, suggesting that protection was against the damaging inflammation, rather than viral titres. This is further exemplified by reduced levels of pro-inflammatory cytokines in *Vim* ^-/-^ mice infected with IAV, including IL-1β, TNF, RANTES (regulated upon activation, normal T cell expressed and presumably secreted), MIP2α (macrophage inflammatory protein 2 alpha), monocyte chemoattractant protein-1 (MCP-1/CCL2), IFN-α and IFN-β (149). This same study also identified three distinct populations of monocyte-derived alveolar macrophages (MoAMs) involved in infiltrating, early and late inflammatory responses to IAV. Compared to WT cells, expression of core pro-inflammatory genes in early phase *Vim* ^-/-^ MoAMs was reduced, while late-stage cells were unaffected by loss of vimentin. Interestingly, 3 distinct clusters of MoAMs were identified in brochoalveolar lavage fluid from patients with severe SARS-CoV-2 pneumonia, the most inflammatory of which also had the highest expression of *VIM* (149).

In addition to its role in NLRP3 inflammasome assembly and activation, vimentin has been shown to interact directly with the LRR domain of NOD2 at the plasma membrane of HEK293 cells (140). Inhibition of this interaction with withaferin A induced relocation of NOD2 from the plasma membrane to the cytosol and abrogated NOD2-dependent NF-κB activation and autophagy in response to treatment of cells with muramyl dipeptide (MDP), a component common to all bacteria (140). This is of potential significance to virus infection, as NOD2 is activated by infection with IAV and promotes IRF3-dependent transactivation of IFN-β (150). Moreover, MDP induces the NOD2-dependent recruitment of inflammatory (Ly6C^high^) monocytes to the lungs of IAV infected mice, coupled with increased type I IFN and MCP-1/CCL2. In both *Nod2* ^-/-^ and *Ccl2* ^-/-^ mice, recruitment of Ly6C^high^ monocytes was impaired and survival of the animals reduced (151).

### Macrophage migration inhibitory factor

Macrophage migration inhibitory factor (MIF) is a pleiotropic pro-inflammatory molecule with multiple proposed roles in immune and non-immune cells. Originally discovered as a lymphokine that inhibits macrophage movement *in vitro*, MIF is now known to be released by most cells and has chemokine function, able to direct the recruitment of lymphocytes, monocytes and neutrophils to sites of inflammation (152). MIF also regulates the release of other cytokines and chemokines, including TNF, MCP-1, IL-8 and IL-1 family members (122, 153, 154) and regulates p53-dependent apoptosis and transcriptional activity (155). However, MIF is an unusual cytokine, possessing tautomerize and thiol-protein oxidoreductase enzymatic activities, although *in vivo* targets for these activities have not yet been found (156). Moreover, precisely how MIF, which does not carry an N-terminal signal peptide required for conventional secretion, is released from cells is not clear. Studies have suggested an unconventional pathway, involving the Golgi complex associated protein p115 and proteins of the ATP binding cassette transporter subfamily 1 (ABCA1) (157, 158), as well as release by dying/necrotic cells (159, 160). Moreover, MIF is induced by, and counteracts the effects of, glucocorticoids, steroid hormones that regulate immune cell function and suppress inflammation (161, 162). MIF inhibitors also have steroid-sparing effects (154). A second MIF family member, D-dopachrome tautomerase (D-DT or MIF-2) has also been characterized, showing structural homology to MIF and similar enzymatic activity, with initial work suggesting that there may be some functional redundancy (163, 164). However, while MIF and D-DT appear to share similar pathogenic roles in multiple sclerosis, they seem to have diverging roles in other diseases, including systemic sclerosis (165).

#### MIF and NLRP3

Studies have demonstrated that *Mif* ^-/-^ mice and their cells secrete less IL-1β and IL-18 in response to infectious organisms than WT controls (166, 167). Moreover, MIF has been shown to drive IL-1β release and disease severity in a mouse model of gout (168). More recently, two studies have highlighted a specific role for MIF in the activation of the NLRP3 inflammasome (122, 169). In one study, inhibition of MIF in macrophages inhibits the release of IL-1β and IL-18 in response to NLRP3-specific stimuli, including the IAV peptide PB1-F2 (122). MIF was required for inflammasome activation and the interaction between NLRP3 and vimentin. Moreover, MIF was found to interact directly, or in complex with, NLRP3, suggesting a direct intracellular role for MIF in NLRP3 inflammasome activation/assembly (122) (**Figure 1**). A second study identified a link between MIF and NLRP3 activation in the response of human monocytes to RNA-containing U1 small nuclear ribonucleoprotein (snRNP) immune complexes (169). Release of MIF by monocytes was increased in response to U1 snRNP immune complexes and concomitant release of IL-1β was inhibited by a small molecule MIF inhibitor, which reduced activation and inhibited the expression of NLRP3 protein and mRNA (169). U1 snRNP immune complexes increased the expression of the cognate MIF receptors CD74 and CD44 (169), which, combined with the previous study, may indicate that MIF can regulate NLRP3-dependent IL-1β release through both intracellular and extracellular activities (122, 169). Extracellular MIF has been proposed to drive NF-κB activation through direct interaction with thioredoxin-interacting protein (TXNIP) (170), which in turn inhibits NF-κB activity. This might be expected to subsequently increase the production of pro-IL-1β, pro-IL-18 and NLRP3, suggesting that MIF inhibitors may abrogate NLRP3 activation through regulation of both NF-κB and NLRP3. However, in our own study, we found no effect of MIF inhibitors on NF-κB activity nor on LPS-induced IL-1β or NLRP3 production (122). Nonetheless, given that MIF is released by necrotic neutrophils and macrophages (159, 160), we can speculate that activation of the NLRP3 inflammasome and subsequent cell death *via* pyroptosis might drive a positive-feedback loop leading to further activation of the NLRP3 inflammasome.

#### MIF and immunity to viruses

While MIF has protective roles to play in immunity against pathogens, including *Salmonella typhimurium*, *Aspergillus fumigatus*, *Trypanosoma cruzi* and *Klebsiella pneumoniae* (167, 171–173), it has pathological, pro-inflammatory roles in animal models of disease, including systemic lupus erythematosus (SLE), multiple sclerosis (MS) and rheumatoid arthritis (RA) (152). MIF has also been implicated in pathological and inflammatory responses to a number of different human viruses, including RSV (121), Ross River virus (174), DENV (123, 124), WNV (130) and IAV (117) (**Table 1**). MIF release by mouse macrophages is increased by replicating RSV *in vitro* and this appears to drive the release of other cytokines including TNF, MCP-1 and IL-10 (121). In the case of DENV, MIF is elevated in infected patients, but there is conflicting data on its correlation with disease severity (125, 126). Nonetheless, inhibition or genetic deletion of MIF has been shown to decrease inflammation and reduce virus replication (123, 175), with one report indicating that increased extracellular MIF might promote DENV replication *in vitro*, by enhancing autophagic flux in Huh-7 cells (175). Serum levels of MIF are raised in patients infected with West Nile virus and patients with high expression MIF polymorphisms were more likely to develop virus associated encephalitis (130).

Serum MIF is similarly increased in patients with IAV, has been associated with disease severity and may have utility as a predictor of disease outcome (176, 177). Intracellular levels of MIF are also raised in human lung epithelial cells infected with IAV (118) and intracellular stores of MIF are released from infected cells when infected cells become necrotic (120). In mouse models of IAV infection, *Mif* ^-/-^ animals have been shown to have reduced lung inflammation, including 10-fold lower levels of IL-1β, reduced viral load and lower mortality (117). Likewise, treatment of infected mice with the MIF inhibitor ISO-1 has been shown to reduce levels of pro-inflammatory cytokine and chemokine mRNA, including IL-1β, in the lungs (178). Conversely, transgenic mice overexpressing MIF in alveolar epithelial cells had more inflammation, higher viral loads and higher mortality (117).

Early studies have also hinted at a pathologic role for MIF in patients infected with SARS-CoV-2. Serum MIF levels were found to be higher in patients with SARS-CoV-2, compared to healthy controls and higher in patients with severe versus moderate disease (114). Similarly, in a study of mechanically ventilated SARS-CoV-2 patients, higher levels of MIF were associated with impaired organ function, increased arterial hypertension and lower 28-day survival (115). In another study in which 65 serum proteins were measured in patients with severe/critical and mild/moderate SARS-CoV-2, along with healthy controls, MIF was the only cytokine raised in both the mild/moderate and severe/critical patients, along with a number of chemokines, growth factors and soluble receptors (116). Interestingly, in this study no difference was observed in MIF levels between the two groups of SARS-CoV-2-infected patients. Levels of IL-18 were significantly higher in critical/hospitalized patients, compared to healthy controls, while IL-1β was not detected in enough patients for analysis (116).

To what extent, if any, MIF regulates host-virus responses through modulation of the NLRP3 inflammasome is not yet understood, although the fact that inhibition or deletion of MIF is almost universally anti-inflammatory, commonly reducing the release of IL-1β, would certainly point to it as a likely pathway of significance. Likewise, the fact that inhibiting MIF abrogates IL-1β and IL-18 release in response to the IAV peptide PB1-F2 (122), further points to regulation of NLRP3 being key to MIFs role in virus-induced hyperinflammation. Thus, targeting MIF, as well as DDX3X and vimentin, potentially offers a novel way of inhibiting NLRP3 clinically.

## Clinical efforts to suppress NLRP3-mediated inflammation

Given NLRP3-mediated inflammation and cytokine storm are associated with disease severity in IAV and SARS-CoV-2 infection (179), suppressing NLRP3 inflammasome hyperactivation and cytokine storm may alleviate acute respiratory distress syndrome (ARDS). As proof-of-principal, MCC950 treatment in a human ACE2 transgenic mouse model (hACE2-tg) quenched levels of inflammatory cytokines and alleviated lung pathology compared to phosphate-buffered saline (PBS)-treated mice (180). Young mice intraperitoneally treated with MCC950 three days post IAV virus infection showed reduced NLRP3 and activated caspase-1, regained weight 8-9 days post-infection and improved survival compared to their PBS-treated IAV-infected counterparts. However, both cohorts exhibited similar lung pathology, suggesting the NLRP3 inflammasome plays a role in recovery from IAV infection (181). A similar study in MCC950-treated IAV-infected adult mice showed that those treated 7 days post-infection had improved survival, however enhanced lethality was observed in mice treated sooner post-infection (182). Therefore, the timing of treatment administration should be considered. MCC950 has been used in phase II clinical trials in patients with RA, in which the activation of the NLRP3 inflammasome plays a role (183), however MCC950 caused liver toxicity (184), sparking the pursuit of NLRP3 inhibitors with enhanced pharmacokinetic properties. To our knowledge only the NLRP3 inhibitor DFV890 (Novartis Pharmaceuticals NCT04382053) has completed phase II clinical trials for efficacy against SARS-CoV-2-induced pneumonia, however DFV890 treatment did not influence disease severity, nor reduce the inflammatory marker serum C-reactive protein. Nonetheless, pharmacological inhibitors of the NLRP3 inflammasome are being avidly pursued, although none are currently FDA-approved.

Quenching the hyperinflammatory response *via* NLRP3 inflammasome inhibitors may be advantageous compared to glucocorticoids, such as dexamethasone, to treat SARS-CoV-2 and IAV infections. While both approaches reduce inflammation, glucocorticoids negatively impact the type I IFN response, which is necessary to restrict viral replication in infected cells and limit viral spread to non-infected cells (185). For this reason, early-stage treatment of glucocorticoids, when the viral load is high, is not recommended. Corticosteroids are also immunosuppressive and have been linked to a rise in life-threatening infections (186–188) and hyperglycemia (189).

## Modulating the NLRP3 modulators

Because it is linked to so many different pathologies, the NLRP3 inflammasome has been a *bona fide* target for drug development for several years, with multiple companies running programs to develop drugs that target this critical inflammatory pathway. Thus, modulators of NLRP3 inflammasome activation, such as DDX3X, vimentin and MIF, may themselves be viable targets for developing agents that block NLRP3-mediated inflammation.

### DDX3X

With its role in NLRP3 inflammasome activation and its conflicting roles in viral replication and suppression, DDX3X is a potentially interesting therapeutic target. For viruses, such as SARS-CoV-2, which utilize DDX3X to service their replication and where infection can progress to life-threatening ARDS, the strategic targeting of DDX3X may combat both viral load and virus-induced inflammation. Intriguingly however, while the DDX3X catalytic inhibitor RK-33 can limit SARS-CoV-2 replication in Vero E6 and Calu-3 cells (70), it did not prevent the cleavage of pro-caspase-1 into the mature p20 subunit following the stimulation of BMDMs with LPS and nigericin (55). Therefore, DDX3X appears to play a protein scaffolding, rather than catalytic role in NLRP3 inflammasome activation. The detailed characterization of the DDX3X-NLRP3 interaction interface may therefore reveal opportunities to disrupt NLRP3 inflammasome activation. Likewise, post-translational modifiers of DDX3X may also prove viable targets. AKT is proposed to phosphorylate DDX3X and in doing so disrupt the DDX3X-NLRP3 interaction (190). The determination of the DDX3X AKT-phosphorylation site/s would validate these results and potentially highlight a unique DDX3X-targeting site. Likewise, other post-translational modifiers of DDX3X, including tripartite motif protein 25 (TRIM25) (39) and TANK binding kinase 1 (TBK1) (43), may influence NLRP3 inflammasome activation but these remain to be explored.

### Vimentin

Given its broad roles in both inflammatory and host-virus responses, vimentin is an attractive target for the development of novel therapeutics. Moreover, because intracellular and extracellular/surface vimentin appear to contribute differently to these roles, the possibility of targeting specific effects is of potential interest. In particular, extracellular vimentin, which can be targeted with anti-vimentin antibodies, has potential for the development of drugs that inhibit virus entry into cells. Targeting of surface vimentin has been shown to inhibit the uptake of SARS-CoV virus-like particles and spike protein by permissive Vero cells (99). Alternatively, small molecule inhibitors, which can enter the cell and access the cytosol, have the potential to disrupt intermediate filament networks, interfering with the intracellular trafficking of viruses, as well as interactions between vimentin and immune signaling pathways, including NLRP3. Withaferin A, a natural withanolide, for example, has been shown to inhibit NLRP3 activation in mouse macrophages (122), as well interfering with the vimentin-NOD interaction. Future strategies to target these specific interactions could lead to the discovery of potent NLRP3-inhibiting drugs.

### MIF

Similar to vimentin, MIF has both intracellular and extracellular functions that are potentially relevant to its role in virus entry and replication, as well as in NLRP3 activation. In a mouse model of IAV infection, treatment of mice with an anti-MIF neutralizing antibody significantly increased survival (117), although whether this is due to effects on NLRP3 activation is unclear. However, treatment of mouse macrophages with the small molecule MIF inhibitor COR123625, has been shown to inhibit IL-1β and IL-18 release in response to the IAV peptide PB1-F2 (122), which is known to activate NLRP3 (34). Of significance, a clinical trial is currently underway to test the efficacy of ibudilast (MN-166) in patients infected with SARS-CoV-2 and at risk of developing ARDS (NCT04429555). Ibudilast is a phosphodiesterase inhibitor approved for the treatment of asthma in Japan that has also been shown to also inhibit MIF tautomerase activity (191). Given that MIF has been shown to interact, either directly or in a complex, with NLRP3 (122), future studies to identify the critical residue/s on the MIF molecule for this interaction could lead to highly specific and potent NLRP3-targeting compounds.

## Conclusions

In the early stages of infection, viral replication is restricted by the type I IFN response and can be aided by treatment with antiviral inhibitors that block viral replication. However, severe viral infections, caused for example by SARS-CoV-2 and IAV, can cause excessive inflammation and develop into life-threatening ARDS. ARDS is currently managed by supportive treatment, such as mechanical ventilation to improve oxygen levels in the blood, however the root cause, hyperinflammation, is typically not pharmacologically treated. In patients with severe SARS-CoV-2, generically quenching the hyperinflammatory response using corticosteroids has shown benefit (192), but may have serious side-effects (193). Alternatively, treating NLRP3-mediated inflammation with specific inhibitors has also shown promise in both SARS-CoV-2- and IAV-infected mouse models and are being pursued by pharmacological companies with great enthusiasm. The identification of new NLRP3 components and accessory molecules, such as DDX3X, vimentin and MIF, which themselves have other regulatory roles in antiviral immunity, reveal the growing complexity of NLRP3 inflammasome regulation. Importantly, it also reveals new opportunities for the development of novel therapeutic strategies to suppress NLRP3-induced inflammation with the potential to reduce disease severity and improve patient outcomes.

## Author contributions

JH and NB: manuscript preparation, including original draft preparation, reviewing, editing and finalizing. All authors contributed to the article and approved the submitted version.

## Funding

This research was funded by the National Health and Medical Research Council (NHMRC) APP1157053.

## Conflict of Interest

The authors declare that the research was conducted in the absence of any commercial or financial relationships that could be construed as a potential conflict of interest.

## Publisher’s note

All claims expressed in this article are solely those of the authors and do not necessarily represent those of their affiliated organizations, or those of the publisher, the editors and the reviewers. Any product that may be evaluated in this article, or claim that may be made by its manufacturer, is not guaranteed or endorsed by the publisher.

## References

[1] Coutard B Valle C de Lamballerie X Canard B Seidah NG Decroly E. The spike glycoprotein of the new coronavirus 2019-nCoV contains a furin-like cleavage site absent in CoV of the same clade. Antiviral Res (2020) 176:104742. doi: 10.1016/j.antiviral.2020.104742 32057769 PMC7114094

[2] Hoffmann M Kleine-Weber H Schroeder S Kruger N Herrler T Erichsen S. SARS-CoV-2 cell entry depends on ACE2 and TMPRSS2 and is blocked by a clinically proven protease inhibitor. Cell (2020) 181(2):271–80.e8. doi: 10.1016/j.cell.2020.02.052 PMC7102627 32142651

[3] Jaimes JA Millet JK Whittaker GR. Proteolytic cleavage of the SARS-CoV-2 spike protein and the role of the novel S1/S2 site. iScience (2020) 23(6):101212. doi: 10.1016/j.isci.2020.101212 32512386 PMC7255728

[4] Huang Y Yang C Xu XF Xu W Liu SW. Structural and functional properties of SARS-CoV-2 spike protein: potential antivirus drug development for COVID-19. Acta Pharmacol Sin (2020) 41(9):1141–9. doi: 10.1038/s41401-020-0485-4 PMC7396720 32747721

[5] Clausen TM Sandoval DR Spliid CB Pihl J Perrett HR Painter CD. SARS-CoV-2 infection depends on cellular heparan sulfate and ACE2. Cell (2020) 183(4):1043–57.e15. doi: 10.1016/j.cell.2020.09.033 PMC7489987 32970989

[6] Wang K Chen W Zhang Z Deng Y Lian JQ Du P. CD147-spike protein is a novel route for SARS-CoV-2 infection to host cells. Signal Transduct Target Ther (2020) 5(1):283. doi: 10.1038/s41392-020-00426-x PMC7714896 33277466

[7] Cantuti-Castelvetri L Ojha R Pedro LD Djannatian M Franz J Kuivanen S. Neuropilin-1 facilitates SARS-CoV-2 cell entry and infectivity. Science (2020) 370(6518):856–60. doi: 10.1126/science.abd2985 PMC7857391 33082293

[8] Amraei R Yin W Napoleon MA Suder EL Berrigan J Zhao Q. CD209L/L-SIGN and CD209/DC-SIGN act as receptors for SARS-CoV-2. ACS Cent Sci (2021) 7(7):1156–65. doi: 10.1021/acscentsci.0c01537 PMC8265543 34341769

[9] Yang ZY Huang Y Ganesh L Leung K Kong WP Schwartz O. pH-dependent entry of severe acute respiratory syndrome coronavirus is mediated by the spike glycoprotein and enhanced by dendritic cell transfer through DC-SIGN. J Virol (2004) 78(11):5642–50. doi: 10.1128/JVI.78.11.5642-5650.2004 PMC415834 15140961

[10] Jeffers SA Tusell SM Gillim-Ross L Hemmila EM Achenbach JE Babcock GJ. CD209L (L-SIGN) is a receptor for severe acute respiratory syndrome coronavirus. Proc Natl Acad Sci U.S.A. (2004) 101(44):15748–53. doi: 10.1073/pnas.0403812101 PMC524836 15496474

[11] Lempp FA Soriaga LB Montiel-Ruiz M Benigni F Noack J Park YJ. Lectins enhance SARS-CoV-2 infection and influence neutralizing antibodies. Nature (2021) 598(7880):342–7. doi: 10.1038/s41586-021-03925-1 34464958

[12] Dou D Revol R Ostbye H Wang H Daniels R. Influenza a virus cell entry, replication, virion assembly and movement. Front Immunol (2018) 9:1581. doi: 10.3389/fimmu.2018.01581 30079062 PMC6062596

[13] Sempere Borau M Stertz S. Entry of influenza a virus into host cells - recent progress and remaining challenges. Curr Opin Virol (2021) 48:23–9. doi: 10.1016/j.coviro.2021.03.001 33838498

[14] Lu Q Liu J Zhao S Gomez Castro MF Laurent-Rolle M Dong J. SARS-CoV-2 exacerbates proinflammatory responses in myeloid cells through c-type lectin receptors and tweety family member 2. Immunity (2021) 54(6):1304–19.e9. doi: 10.1016/j.immuni.2021.05.006 PMC8106883 34048708

[15] Khan S Shafiei MS Longoria C Schoggins JW Savani RC Zaki H. SARS-CoV-2 spike protein induces inflammation *via* TLR2-dependent activation of the NF-kappaB pathway. Elife (2021) 10:e68563. doi: 10.7554/eLife.68563 34866574 PMC8709575

[16] Naqvi I Giroux N Olson L Morrison SA Llanga T Akinade TO. DAMPs/PAMPs induce monocytic TLR activation and tolerance in COVID-19 patients; nucleic acid binding scavengers can counteract such TLR agonists. Biomaterials (2022) 283:121393. doi: 10.1016/j.biomaterials.2022.121393 35349874 PMC8797062

[17] Kouwaki T Nishimura T Wang G Oshiumi H. RIG-I-Like receptor-mediated recognition of viral genomic RNA of severe acute respiratory syndrome coronavirus-2 and viral escape from the host innate immune responses. Front Immunol (2021) 12:700926. doi: 10.3389/fimmu.2021.700926 34249006 PMC8267574

[18] Chyuan IT Tzeng HT Chen JY. Signaling pathways of type I and type III interferons and targeted therapies in systemic lupus erythematosus. Cells (2019) 8(9):963. doi: 10.3390/cells8090963 PMC6769759 31450787

[19] Schindler C Levy DE Decker T. JAK-STAT signaling: from interferons to cytokines. J Biol Chem (2007) 282(28):20059–63. doi: 10.1074/jbc.R700016200 17502367

[20] Lupfer C Malik A Kanneganti TD. Inflammasome control of viral infection. Curr Opin Virol (2015) 12:38–46. doi: 10.1016/j.coviro.2015.02.007 PMC4470791 25771504

[21] Swanson KV Deng M Ting JP. The NLRP3 inflammasome: molecular activation and regulation to therapeutics. Nat Rev Immunol (2019) 19(8):477–89. doi: 10.1038/s41577-019-0165-0 PMC7807242 31036962

[22] Hafner-Bratkovic I Susjan P Lainscek D Tapia-Abellan A Cerovic K Kadunc L. NLRP3 lacking the leucine-rich repeat domain can be fully activated *via* the canonical inflammasome pathway. Nat Commun (2018) 9(1):5182. doi: 10.1038/s41467-018-07573-4 PMC6281599 30518920

[23] He Y Hara H Nunez G. Mechanism and regulation of NLRP3 inflammasome activation. Trends Biochem Sci (2016) 41(12):1012–21. doi: 10.1016/j.tibs.2016.09.002 PMC5123939 27669650

[24] Gritsenko A Yu S Martin-Sanchez F Diaz-Del-Olmo I Nichols EM Davis DM. Priming is dispensable for NLRP3 inflammasome activation in human monocytes. In Vitro. Front Immunol (2020) 11:565924. doi: 10.3389/fimmu.2020.565924 33101286 PMC7555430

[25] Munoz-Planillo R Kuffa P Martinez-Colon G Smith BL Rajendiran TM Nunez G. K(+) efflux is the common trigger of NLRP3 inflammasome activation by bacterial toxins and particulate matter. Immunity (2013) 38(6):1142–53. doi: 10.1016/j.immuni.2013.05.016 PMC3730833 23809161

[26] Aglietti RA Estevez A Gupta A Ramirez MG Liu PS Kayagaki N. GsdmD p30 elicited by caspase-11 during pyroptosis forms pores in membranes. Proc Natl Acad Sci U.S.A. (2016) 113(28):7858–63. doi: 10.1073/pnas.1607769113 PMC4948338 27339137

[27] Ding J Wang K Liu W She Y Sun Q Shi J. Pore-forming activity and structural autoinhibition of the gasdermin family. Nature (2016) 535(7610):111–6. doi: 10.1038/nature18590 27281216

[28] Liu X Zhang Z Ruan J Pan Y Magupalli VG Wu H. Inflammasome-activated gasdermin d causes pyroptosis by forming membrane pores. Nature (2016) 535(7610):153–8. doi: 10.1038/nature18629 PMC5539988 27383986

[29] Mulvihill E Sborgi L Mari SA Pfreundschuh M Hiller S Muller DJ. Mechanism of membrane pore formation by human gasdermin-d. EMBO J (2018) 37(14):e98321. doi: 10.15252/embj.201798321 29898893 PMC6043855

[30] Sborgi L Ruhl S Mulvihill E Pipercevic J Heilig R Stahlberg H. GSDMD membrane pore formation constitutes the mechanism of pyroptotic cell death. EMBO J (2016) 35(16):1766–78. doi: 10.15252/embj.201694696 PMC5010048 27418190

[31] da Costa LS Outlioua A Anginot A Akarid K Arnoult D. RNA Viruses promote activation of the NLRP3 inflammasome through cytopathogenic effect-induced potassium efflux. Cell Death Dis (2019) 10(5):346. doi: 10.1038/s41419-019-1579-0 PMC6483999 31024004

[32] Allen IC Scull MA Moore CB Holl EK McElvania-TeKippe E Taxman DJ. The NLRP3 inflammasome mediates *in vivo* innate immunity to influenza a virus through recognition of viral RNA. Immunity (2009) 30(4):556–65. doi: 10.1016/j.immuni.2009.02.005 PMC2803103 19362020

[33] Ichinohe T Pang IK Iwasaki A. Influenza virus activates inflammasomes *via* its intracellular M2 ion channel. Nat Immunol (2010) 11(5):404–10. doi: 10.1038/ni.1861 PMC2857582 20383149

[34] McAuley JL Tate MD MacKenzie-Kludas CJ Pinar A Zeng W Stutz A. Activation of the NLRP3 inflammasome by IAV virulence protein PB1-F2 contributes to severe pathophysiology and disease. PloS Pathog (2013) 9(5):e1003392. doi: 10.1371/journal.ppat.1003392 23737748 PMC3667782

[35] Pan P Shen M Yu Z Ge W Chen K Tian M. SARS-CoV-2 n protein promotes NLRP3 inflammasome activation to induce hyperinflammation. Nat Commun (2021) 12(1):4664. doi: 10.1038/s41467-021-25015-6 PMC8329225 34341353

[36] Sun X Liu Y Huang Z Xu W Hu W Yi L. SARS-CoV-2 non-structural protein 6 triggers NLRP3-dependent pyroptosis by targeting ATP6AP1. Cell Death Differ (2022) 29(6):1240–54. doi: 10.1038/s41418-021-00916-7 PMC9177730 34997207

[37] Cheung PH Ye ZW Lee TT Chen H Chan CP Jin DY. PB1-F2 protein of highly pathogenic influenza a (H7N9) virus selectively suppresses RNA-induced NLRP3 inflammasome activation through inhibition of MAVS-NLRP3 interaction. J Leukoc Biol (2020) 108(5):1655–63. doi: 10.1002/JLB.4AB0420-694R 32386456

[38] Yoshizumi T Ichinohe T Sasaki O Otera H Kawabata S Mihara K. Influenza a virus protein PB1-F2 translocates into mitochondria *via* Tom40 channels and impairs innate immunity. Nat Commun (2014) 5:4713. doi: 10.1038/ncomms5713 25140902

[39] Atkinson SC Heaton SM Audsley MD Kleifeld O Borg NA. TRIM25 and DEAD-box RNA helicase DDX3X cooperate to regulate RIG-I-Mediated antiviral immunity. Int J Mol Sci (2021) 22(16):9094. doi: 10.3390/ijms22169094 34445801 PMC8396550

[40] Gu L Fullam A Brennan R Schroder M. Human DEAD box helicase 3 couples IkappaB kinase epsilon to interferon regulatory factor 3 activation. Mol Cell Biol (2013) 33(10):2004–15. doi: 10.1128/MCB.01603-12 PMC3647972 23478265

[41] Oshiumi H Sakai K Matsumoto M Seya T. DEAD/H BOX 3 (DDX3) helicase binds the RIG-I adaptor IPS-1 to up-regulate IFN-beta-inducing potential. Eur J Immunol (2010) 40(4):940–8. doi: 10.1002/eji.200940203 20127681

[42] Schroder M Baran M Bowie AG. Viral targeting of DEAD box protein 3 reveals its role in TBK1/IKKepsilon-mediated IRF activation. EMBO J (2008) 27(15):2147–57. doi: 10.1038/emboj.2008.143 PMC2516890 18636090

[43] Soulat D Burckstummer T Westermayer S Goncalves A Bauch A Stefanovic A. The DEAD-box helicase DDX3X is a critical component of the TANK-binding kinase 1-dependent innate immune response. EMBO J (2008) 27(15):2135–46. doi: 10.1038/emboj.2008.126 PMC2453059 18583960

[44] Shih JW Tsai TY Chao CH Wu Lee YH. Candidate tumor suppressor DDX3 RNA helicase specifically represses cap-dependent translation by acting as an eIF4E inhibitory protein. Oncogene (2008) 27(5):700–14. doi: 10.1038/sj.onc.1210687 17667941

[45] Shih JW Wang WT Tsai TY Kuo CY Li HK Wu Lee YH. Critical roles of RNA helicase DDX3 and its interactions with eIF4E/PABP1 in stress granule assembly and stress response. Biochem J (2012) 441(1):119–29. doi: 10.1042/BJ20110739 21883093

[46] Jain S Wheeler JR Walters RW Agrawal A Barsic A Parker R. ATPase-modulated stress granules contain a diverse proteome and substructure. Cell (2016) 164(3):487–98. doi: 10.1016/j.cell.2015.12.038 PMC4733397 26777405

[47] Protter DSW Parker R. Principles and properties of stress granules. Trends Cell Biol (2016) 26(9):668–79. doi: 10.1016/j.tcb.2016.05.004 PMC4993645 27289443

[48] Mo J Liang H Su C Li P Chen J Zhang B. DDX3X: structure, physiologic functions and cancer. Mol Cancer (2021) 20(1):38. doi: 10.1186/s12943-021-01325-7 PMC7903766 33627125

[49] Schroder M. Viruses and the human DEAD-box helicase DDX3: inhibition or exploitation? Biochem Soc Trans (2011) 39(2):679–83. doi: 10.1042/BST0390679 21428961

[50] Tantravedi S Vesuna F Winnard PT Jr. Van Voss MRH Van Diest PJ Raman V. Role of DDX3 in the pathogenesis of inflammatory bowel disease. Oncotarget (2017) 8(70):115280–9. doi: 10.18632/oncotarget.23323 PMC5777771 29383159

[51] Venkataramanan S Gadek M Calviello L Wilkins K Floor SN. DDX3X and DDX3Y are redundant in protein synthesis. RNA (2021) 27(12):1577–88. doi: 10.1261/rna.078926.121 PMC8594478 34535544

[52] Szappanos D Tschismarov R Perlot T Westermayer S Fischer K Platanitis E. The RNA helicase DDX3X is an essential mediator of innate antimicrobial immunity. PloS Pathog (2018) 14(11):e1007397. doi: 10.1371/journal.ppat.1007397 30475900 PMC6283616

[53] Gal-Oz ST Maier B Yoshida H Seddu K Elbaz N Czysz C. ImmGen report: sexual dimorphism in the immune system transcriptome. Nat Commun (2019) 10(1):4295. doi: 10.1038/s41467-019-12348-6 PMC6754408 31541153

[54] Feng D Guo L Liu J Song Y Ma X Hu H. DDX3X deficiency alleviates LPS-induced H9c2 cardiomyocytes pyroptosis by suppressing activation of NLRP3 inflammasome. Exp Ther Med (2021) 22(6):1389. doi: 10.3892/etm.2021.10825 PMC8506920 34650637

[55] Samir P Kesavardhana S Patmore DM Gingras S Malireddi RKS Karki R. DDX3X acts as a live-or-die checkpoint in stressed cells by regulating NLRP3 inflammasome. Nature (2019) 573(7775):590–4. doi: 10.1038/s41586-019-1551-2 PMC6980284 31511697

[56] Kesavardhana S Samir P Zheng M Malireddi RKS Karki R Sharma BR. DDX3X coordinates host defense against influenza virus by activating the NLRP3 inflammasome and type I interferon response. J Biol Chem (2021) 296:100579. doi: 10.1016/j.jbc.2021.100579 33766561 PMC8081917

[57] Ellwanger K Becker E Kienes I Sowa A Postma Y Cardona Gloria Y. The NLR family pyrin domain-containing 11 protein contributes to the regulation of inflammatory signaling. J Biol Chem (2018) 293(8):2701–10. doi: 10.1074/jbc.RA117.000152 PMC5827450 29301940

[58] Qin Y Su Z Wu Y Wu C Jin S Xie W. NLRP11 disrupts MAVS signalosome to inhibit type I interferon signaling and virus-induced apoptosis. EMBO Rep (2017) 18(12):2160–71. doi: 10.15252/embr.201744480 PMC5709773 29097393

[59] Wu C Su Z Lin M Ou J Zhao W Cui J. NLRP11 attenuates toll-like receptor signalling by targeting TRAF6 for degradation *via* the ubiquitin ligase RNF19A. Nat Commun (2017) 8(1):1977. doi: 10.1038/s41467-017-02073-3 PMC5719394 29215004

[60] Gangopadhyay A Devi S Tenguria S Carriere J Nguyen H Jager E. NLRP3 licenses NLRP11 for inflammasome activation in human macrophages. Nat Immunol (2022) 23(6):892–903. doi: 10.1038/s41590-022-01220-3 PMC9174058 35624206

[61] Oda S Schroder M Khan AR. Structural basis for targeting of human RNA helicase DDX3 by poxvirus protein K7. Structure (2009) 17(11):1528–37. doi: 10.1016/j.str.2009.09.005 19913487

[62] Ishaq M Hu J Wu X Fu Q Yang Y Liu Q. Knockdown of cellular RNA helicase DDX3 by short hairpin RNAs suppresses HIV-1 viral replication without inducing apoptosis. Mol Biotechnol (2008) 39(3):231–8. doi: 10.1007/s12033-008-9040-0 18259889

[63] Yedavalli VS Neuveut C Chi YH Kleiman L Jeang KT. Requirement of DDX3 DEAD box RNA helicase for HIV-1 rev-RRE export function. Cell (2004) 119(3):381–92. doi: 10.1016/j.cell.2004.09.029 15507209

[64] Maga G Falchi F Radi M Botta L Casaluce G Bernardini M. Toward the discovery of novel anti-HIV drugs. second-generation inhibitors of the cellular ATPase DDX3 with improved anti-HIV activity. ChemMedChem (2011) 6(8):1371–89. doi: 10.1002/cmdc.201100166 21698775

[65] Radi M Botta M Falchi F Maga G Baldanti F Paolucci S. Inventor; compounds with DDX3 inhibitory activity and uses thereof patent PCT/IB2010/054475. (2011).

[66] Radi M Falchi F Garbelli A Samuele A Bernardo V Paolucci S. Discovery of the first small molecule inhibitor of human DDX3 specifically designed to target the RNA binding site: towards the next generation HIV-1 inhibitors. Bioorg Med Chem Lett (2012) 22(5):2094–8. doi: 10.1016/j.bmcl.2011.12.135 22300661

[67] Yedavalli VS Zhang N Cai H Zhang P Starost MF Hosmane RS. Ring expanded nucleoside analogues inhibit RNA helicase and intracellular human immunodeficiency virus type 1 replication. J Med Chem (2008) 51(16):5043–51. doi: 10.1021/jm800332m PMC2537995 18680273

[68] Meyerhans A Martinez de la Sierra M Brai A Fazi R Tintori C Botta M. Human helicase DDX3X inhibitors as therapeutic agents patent PCT/EP2016/052990. (2016).

[69] Vesuna F Akhrymuk I Smith A Winnard PT Lin SC Scharpf R. RK-33, a small molecule inhibitor of host RNA helicase DDX3, suppresses multiple variants of SARS-CoV-2. bioRxiv (2022). doi: 10.1101/2022.02.28.482334 PMC9453862 36090095

[70] Ciccosanti F Di Rienzo M Romagnoli A Colavita F Refolo G Castilletti C. Proteomic analysis identifies the RNA helicase DDX3X as a host target against SARS-CoV-2 infection. Antiviral Res (2021) 190:105064. doi: 10.1016/j.antiviral.2021.105064 33781803 PMC7997689

[71] Thulasi Raman SN Liu G Pyo HM Cui YC Xu F Ayalew LE. DDX3 interacts with influenza a virus NS1 and NP proteins and exerts antiviral function through regulation of stress granule formation. J Virol (2016) 90(7):3661–75. doi: 10.1128/JVI.03010-15 PMC4794679 26792746

[72] Chung WC Kang HR Yoon H Kang SJ Ting JP Song MJ. Influenza a virus NS1 protein inhibits the NLRP3 inflammasome. PloS One (2015) 10(5):e0126456. doi: 10.1371/journal.pone.0126456 25978411 PMC4433236

[73] Moriyama M Chen IY Kawaguchi A Koshiba T Nagata K Takeyama H. The RNA- and TRIM25-binding domains of influenza virus NS1 protein are essential for suppression of NLRP3 inflammasome-mediated interleukin-1beta secretion. J Virol (2016) 90(8):4105–14. doi: 10.1128/JVI.00120-16 PMC4810543 26865721

[74] Park HS Liu G Thulasi Raman SN Landreth SL Liu Q Zhou Y. NS1 protein of 2009 pandemic influenza a virus inhibits porcine NLRP3 inflammasome-mediated interleukin-1 beta production by suppressing ASC ubiquitination. J Virol (2018) 92(8):e00022-18. doi: 10.1128/JVI.00022-18 29386291 PMC5874408

[75] Stasakova J Ferko B Kittel C Sereinig S Romanova J Katinger H. Influenza a mutant viruses with altered NS1 protein function provoke caspase-1 activation in primary human macrophages, resulting in fast apoptosis and release of high levels of interleukins 1beta and 18. J Gen Virol (2005) 86(Pt 1):185–95. doi: 10.1099/vir.0.80422-0 15604446

[76] Easton A Gould P Marsh A. Inventor; use of DDX3X inhibitors for the treatment of pneumovirus infections patent PCT/GB2015/050724. (2012).

[77] Yang SNY Atkinson SC Audsley MD Heaton SM Jans DA Borg NA. RK-33 is a broad-spectrum antiviral agent that targets DEAD-box RNA helicase DDX3X. Cells (2020) 9(1):170. doi: 10.3390/cells9010170 PMC7016805 31936642

[78] Khadka S Vangeloff AD Zhang C Siddavatam P Heaton NS Wang L. A physical interaction network of dengue virus and human proteins. Mol Cell Proteomics (2011) 10(12):M111.012187. doi: 10.1074/mcp.M111.012187 PMC3237087 21911577

[79] Kumar R Singh N Abdin MZ Patel AH Medigeshi GR. Dengue virus capsid interacts with DDX3X-a potential mechanism for suppression of antiviral functions in dengue infection. Front Cell Infect Microbiol (2017) 7:542. doi: 10.3389/fcimb.2017.00542 29387631 PMC5776122

[80] Brai A Fazi R Tintori C Zamperini C Bugli F Sanguinetti M. Human DDX3 protein is a valuable target to develop broad spectrum antiviral agents. Proc Natl Acad Sci USA (2016) 113(19):5388–93. doi: 10.1073/pnas.1522987113 PMC4868442 27118832

[81] Brai A Boccuto A Monti M Marchi S Vicenti I Saladini F. Exploring the implication of DDX3X in DENV infection: Discovery of the first-in-Class DDX3X fluorescent inhibitor. ACS Med Chem Lett (2020) 11(5):956–62. doi: 10.1021/acsmedchemlett.9b00681 PMC7236276 32435411

[82] Li G Feng T Pan W Shi X Dai J. DEAD-box RNA helicase DDX3X inhibits DENV replication *via* regulating type one interferon pathway. Biochem Biophys Res Commun (2015) 456(1):327–32. doi: 10.1016/j.bbrc.2014.11.080 25437271

[83] Nelson C Mrozowich T Gemmill DL Park SM Patel TR. Human DDX3X unwinds Japanese encephalitis and zika viral 5' terminal regions. Int J Mol Sci (2021) 22(1):413. doi: 10.3390/ijms22010413 PMC7795613 33401776

[84] Brai A Martelli F Riva V Garbelli A Fazi R Zamperini C. DDX3X helicase inhibitors as a new strategy to fight the West Nile virus infection. J Med Chem (2019) 62(5):2333–47. doi: 10.1021/acs.jmedchem.8b01403 30721061

[85] Brai A Martelli F Riva V Garbelli A Fazi R Zamperini C. DDX3X helicase inhibitors as a new strategy to fight the West Nile virus infection. J Med Chem (2019) 2333–47. doi: 10.1021/acs.jmedchem.8b01403 30721061

[86] Li C Ge LL Li PP Wang Y Dai JJ Sun MX. Cellular DDX3 regulates Japanese encephalitis virus replication by interacting with viral un-translated regions. Virology (2014) 449:70–81. doi: 10.1016/j.virol.2013.11.008 PMC7111930 24418539

[87] Wang H Kim S Ryu WS. DDX3 DEAD-box RNA helicase inhibits hepatitis b virus reverse transcription by incorporation into nucleocapsids. J Virol (2009) 83(11):5815–24. doi: 10.1128/JVI.00011-09 PMC2681949 19297497

[88] Ko C Lee S Windisch MP Ryu WS. DDX3 DEAD-box RNA helicase is a host factor that restricts hepatitis b virus replication at the transcriptional level. J Virol (2014) 88(23):13689–98. doi: 10.1128/JVI.02035-14 PMC4248967 25231298

[89] Li Q Pene V Krishnamurthy S Cha H Liang TJ. Hepatitis c virus infection activates an innate pathway involving IKK-alpha in lipogenesis and viral assembly. Nat Med (2013) 19(6):722–9. doi: 10.1038/nm.3190 PMC3676727 23708292

[90] Pene V Li Q Sodroski C Hsu CS Liang TJ. Dynamic interaction of stress granules, DDX3X, and IKK-alpha mediates multiple functions in hepatitis c virus infection. J Virol (2015) 89(10):5462–77. doi: 10.1128/JVI.03197-14 PMC4442532 25740981

[91] Ariumi Y Kuroki M Abe K Dansako H Ikeda M Wakita T. DDX3 DEAD-box RNA helicase is required for hepatitis c virus RNA replication. J Virol (2007) 81(24):13922–6. doi: 10.1128/JVI.01517-07 PMC2168844 17855521

[92] Randall G Panis M Cooper JD Tellinghuisen TL Sukhodolets KE Pfeffer S. Cellular cofactors affecting hepatitis c virus infection and replication. Proc Natl Acad Sci U.S.A. (2007) 104(31):12884–9. doi: 10.1073/pnas.0704894104 PMC1937561 17616579

[93] Angus AG Dalrymple D Boulant S McGivern DR Clayton RF Scott MJ. Requirement of cellular DDX3 for hepatitis c virus replication is unrelated to its interaction with the viral core protein. J Gen Virol (2010) 91(Pt 1):122–32. doi: 10.1099/vir.0.015909-0 PMC2885062 19793905

[94] Mamiya N Worman HJ. Hepatitis c virus core protein binds to a DEAD box RNA helicase. J Biol Chem (1999) 274(22):15751–6. doi: 10.1074/jbc.274.22.15751 10336476

[95] Oshiumi H Ikeda M Matsumoto M Watanabe A Takeuchi O Akira S. Hepatitis c virus core protein abrogates the DDX3 function that enhances IPS-1-mediated IFN-beta induction. PloS One (2010) 5(12):e14258. doi: 10.1371/journal.pone.0014258 21170385 PMC2999533

[96] Owsianka AM Patel AH. Hepatitis c virus core protein interacts with a human DEAD box protein DDX3. Virology (1999) 257(2):330–40. doi: 10.1006/viro.1999.9659 10329544

[97] Sato S Fukasawa M Yamakawa Y Natsume T Suzuki T Shoji I. Proteomic profiling of lipid droplet proteins in hepatoma cell lines expressing hepatitis c virus core protein. J Biochem (2006) 139(5):921–30. doi: 10.1093/jb/mvj104 16751600

[98] Fernandez-Ortega C Ramirez A Casillas D Paneque T Ubieta R Dubed M. Identification of vimentin as a potential therapeutic target against HIV infection. Viruses (2016) 8(6):14. doi: 10.3390/v8060098 PMC4926169 27314381

[99] Yu YT Chien SC Chen IY Lai CT Tsay YG Chang SC. Surface vimentin is critical for the cell entry of SARS-CoV. J BioMed Sci (2016) 23:14. doi: 10.1186/s12929-016-0234-7 PMC4724099 26801988

[100] Amraei R Xia C Olejnik J White MR Napoleon MA Lotfollahzadeh S. Extracellular vimentin is an attachment factor that facilitates SARS-CoV-2 entry into human endothelial cells. Proc Natl Acad Sci USA (2022) 119(6):e2113874119. doi: 10.1073/pnas.2113874119 35078919 PMC8833221

[101] Lalioti V Gonzalez-Sanz S Lois-Bermejo I Gonzalez-Jimenez P Viedma-Poyatos A Merino A. Cell surface detection of vimentin, ACE2 and SARS-CoV-2 spike proteins reveals selective colocalization at primary cilia. Sci Rep (2022) 12(1):7063. doi: 10.1038/s41598-022-11248-y PMC9052736 35487944

[102] Li Z Wu J Zhou J Yuan B Chen J Wu W. A vimentin-targeting oral compound with host-directed antiviral and anti-inflammatory actions addresses multiple features of COVID-19 and related diseases. mBio (2021) 12(5):e0254221. doi: 10.1128/mBio.02542-21 34634931 PMC8510534

[103] Wu W Pante N. Vimentin plays a role in the release of the influenza a viral genome from endosomes. Virology (2016) 497:41–52. doi: 10.1016/j.virol.2016.06.021 27423069

[104] Huang SY Huang CH Chen CJ Chen TW Lin CY Lin YT. Novel role for miR-1290 in host species specificity of influenza a virus. Mol Ther Nucleic Acids (2019) 17:10–23. doi: 10.1016/j.omtn.2019.04.028 PMC6554369 31173947

[105] Yu J Li X Zhou D Liu X He X Huang SH. Vimentin inhibits dengue virus type 2 invasion of the blood-brain barrier. Front Cell Infect Microbiol (2022) 12:868407. doi: 10.3389/fcimb.2022.868407 35433510 PMC9005901

[106] Teo CS Chu JJ. Cellular vimentin regulates construction of dengue virus replication complexes through interaction with NS4A protein. J Virol (2014) 88(4):1897–913. doi: 10.1128/JVI.01249-13 PMC3911532 24284321

[107] Risco C Rodriguez JR Lopez-Iglesias C Carrascosa JL Esteban M Rodriguez D. Endoplasmic reticulum-golgi intermediate compartment membranes and vimentin filaments participate in vaccinia virus assembly. J Virol (2002) 76(4):1839–55. doi: 10.1128/jvi.76.4.1839-1855.2002 PMC135913 11799179

[108] Miller MS Hertel L. Onset of human cytomegalovirus replication in fibroblasts requires the presence of an intact vimentin cytoskeleton. J Virol (2009) 83(14):7015–28. doi: 10.1128/JVI.00398-09 PMC2704777 19403668

[109] Turkki P Laajala M Flodstrom-Tullberg M Marjomaki V. Human enterovirus group b viruses rely on vimentin dynamics for efficient processing of viral nonstructural proteins. J Virol (2020) 94(2):e01393-19. doi: 10.1128/JVI.01393-19 31619557 PMC6955253

[110] Schafer G Graham LM Lang DM Blumenthal MJ Bergant Marusic M Katz AA. Vimentin modulates infectious internalization of human papillomavirus 16 pseudovirions. J Virol (2017) 91(16):e00307-17. doi: 10.1128/JVI.00307-17 28566373 PMC5533935

[111] Nitahara-Kasahara Y Fukasawa M Shinkai-Ouchi F Sato S Suzuki T Murakami K. Cellular vimentin content regulates the protein level of hepatitis c virus core protein and the hepatitis c virus production in cultured cells. Virology (2009) 383(2):319–27. doi: 10.1016/j.virol.2008.10.009 19013628

[112] Ma X Ling Y Li P Sun P Cao Y Bai X. Cellular vimentin interacts with foot-and-Mouth disease virus nonstructural protein 3A and negatively modulates viral replication. J Virol (2020) 94(16):e00273-20. doi: 10.1128/JVI.00273-20 32493819 PMC7394891

[113] Regis EG Barreto-de-Souza V Morgado MG Bozza MT Leng L Bucala R. Elevated levels of macrophage migration inhibitory factor (MIF) in the plasma of HIV-1-infected patients and in HIV-1-infected cell cultures: a relevant role on viral replication. Virology (2010) 399(1):31–8. doi: 10.1016/j.virol.2009.12.018 PMC3140709 20085845

[114] Aksakal A Kerget B Kerget F Askin S. Evaluation of the relationship between macrophage migration inhibitory factor level and clinical course in patients with COVID-19 pneumonia. J Med Virol (2021) 93(12):6519–24. doi: 10.1002/jmv.27189 PMC8426684 34241898

[115] Bleilevens C Soppert J Hoffmann A Breuer T Bernhagen J Martin L. Macrophage migration inhibitory factor (MIF) plasma concentration in critically ill COVID-19 patients: A prospective observational study. Diagn (Basel) (2021) 11(2):332. doi: 10.3390/diagnostics11020332 PMC7922575 33671433

[116] Syed F Li W Relich RF Russell PM Zhang S Zimmerman MK. Excessive matrix metalloproteinase-1 and hyperactivation of endothelial cells occurred in COVID-19 patients and were associated with the severity of COVID-19. J Infect Dis (2021) 224(1):60–9. doi: 10.1093/infdis/jiab167 PMC8083685 33885811

[117] Smith CA Tyrell DJ Kulkarni UA Wood S Leng L Zemans RL. Macrophage migration inhibitory factor enhances influenza-associated mortality in mice. JCI Insight (2019) 4(13):e128034. doi: 10.1172/jci.insight.128034 PMC6629144 31292300

[118] Zhao H Yang J Li K Ding X Lin R Ma Y. Proteomic analysis at the subcellular level for host targets against influenza a virus (H1N1). Antiviral Res (2013) 100(3):673–87. doi: 10.1016/j.antiviral.2013.10.005 24161511

[119] Guan ZH Zhang ML Hou PL Duan M Cui YM Wang XR. Identification of cellular proteins interacting with influenza a virus PB1-F2 protein. Acta Virol (2012) 56(3):199–207. doi: 10.4149/av_2012_03_199 23043599

[120] Arndt U Wennemuth G Barth P Nain M Al-Abed Y Meinhardt A. Release of macrophage migration inhibitory factor and CXCL8/interleukin-8 from lung epithelial cells rendered necrotic by influenza a virus infection. J Virol (2002) 76(18):9298–306. doi: 10.1128/jvi.76.18.9298-9306.2002 PMC136427 12186913

[121] de Souza GF Muraro SP Santos LD Monteiro APT da Silva AG de Souza APD. Macrophage migration inhibitory factor (MIF) controls cytokine release during respiratory syncytial virus infection in macrophages. Inflammation Res (2019) 68(6):481–91. doi: 10.1007/s00011-019-01233-z 30944975

[122] Lang T Lee JPW Elgass K Pinar AA Tate MD Aitken EH. Macrophage migration inhibitory factor is required for NLRP3 inflammasome activation. Nat Commun (2018) 9(1):2223. doi: 10.1038/s41467-018-04581-2 PMC5993818 29884801

[123] Assuncao-Miranda I Amaral FA Bozza FA Fagundes CT Sousa LP Souza DG. Contribution of macrophage migration inhibitory factor to the pathogenesis of dengue virus infection. FASEB J (2010) 24(1):218–28. doi: 10.1096/fj.09-139469 19776337

[124] Chuang YC Chen HR Yeh TM. Pathogenic roles of macrophage migration inhibitory factor during dengue virus infection. Mediators Inflammation (2015) 2015:547094. doi: 10.1155/2015/547094 PMC4363636 25821355

[125] Chen LC Lei HY Liu CC Shiesh SC Chen SH Liu HS. Correlation of serum levels of macrophage migration inhibitory factor with disease severity and clinical outcome in dengue patients. Am J Trop Med Hyg (2006) 74(1):142–7. doi: 10.4269/ajtmh.2006.74.142 16407359

[126] Oliveira R Cordeiro MT Moura P Baptista Filho PNB Braga-Neto UM Marques ETAJ. Serum cytokine/chemokine profiles in patients with dengue fever (DF) and dengue hemorrhagic fever (FHD) by using protein array. J Clin Virol (2017) 89:39–45. doi: 10.1016/j.jcv.2017.02.007 28242509

[127] Chen HR Chuang YC Lin YS Liu HS Liu CC Perng GC. Dengue virus nonstructural protein 1 induces vascular leakage through macrophage migration inhibitory factor and autophagy. PloS Negl Trop Dis (2016) 10(7):e0004828. doi: 10.1371/journal.pntd.0004828 27409803 PMC4943727

[128] Chuang YC Lei HY Liu HS Lin YS Fu TF Yeh TM. Macrophage migration inhibitory factor induced by dengue virus infection increases vascular permeability. Cytokine (2011) 54(2):222–31. doi: 10.1016/j.cyto.2011.01.013 21320786

[129] Lang J Cheng Y Rolfe A Hammack C Vera D Kyle K. An hPSC-derived tissue-resident macrophage model reveals differential responses of macrophages to ZIKV and DENV infection. Stem Cell Rep (2018) 11(2):348–62. doi: 10.1016/j.stemcr.2018.06.006 PMC6092684 29983385

[130] Das R Loughran K Murchison C Qian F Leng L Song Y. Association between high expression macrophage migration inhibitory factor (MIF) alleles and West Nile virus encephalitis. Cytokine (2016) 78:51–4. doi: 10.1016/j.cyto.2015.11.021 PMC4696904 26638028

[131] Arjona A Foellmer HG Town T Leng L McDonald C Wang T. Abrogation of macrophage migration inhibitory factor decreases West Nile virus lethality by limiting viral neuroinvasion. J Clin Invest (2007) 117(10):3059–66. doi: 10.1172/JCI32218 PMC1994625 17909632

[132] Constant O Barthelemy J Nagy A Salinas S Simonin Y. West Nile Virus Neuroinfection in Humans: Peripheral Biomarkers of Neuroinflammation and Neuronal Damage. Viruses (2022) 14(4):756. doi: 10.3390/v14040756 35458486 PMC9027124

[133] Ruggieri A Dazert E Metz P Hofmann S Bergeest JP Mazur J. Dynamic oscillation of translation and stress granule formation mark the cellular response to virus infection. Cell Host Microbe (2012) 12(1):71–85. doi: 10.1016/j.chom.2012.05.013 PMC3873964 22817989

[134] Ramos I Stamatakis K Oeste CL Perez-Sala D. Vimentin as a multifaceted player and potential therapeutic target in viral infections. Int J Mol Sci (2020) 21(13):4675. doi: 10.3390/ijms21134675 PMC7370124 32630064

[135] Frescas D Roux CM Aygun-Sunar S Gleiberman AS Krasnov P Kurnasov OV. Senescent cells expose and secrete an oxidized form of membrane-bound vimentin as revealed by a natural polyreactive antibody. Proc Natl Acad Sci USA (2017) 114(9):E1668–E77. doi: 10.1073/pnas.1614661114 PMC5338544 28193858

[136] Ivaska J Pallari HM Nevo J Eriksson JE. Novel functions of vimentin in cell adhesion, migration, and signaling. Exp Cell Res (2007) 313(10):2050–62. doi: 10.1016/j.yexcr.2007.03.040 17512929

[137] Janciauskiene S Tumpara S Wiese M Wrenger S Vijayan V Gueler F. Alpha1-antitrypsin binds hemin and prevents oxidative activation of human neutrophils: putative pathophysiological significance. J Leukoc Biol (2017) 102(4):1127–41. doi: 10.1189/jlb.3A0317-124R 28716864

[138] Mor-Vaknin N Punturieri A Sitwala K Markovitz DM. Vimentin is secreted by activated macrophages. Nat Cell Biol (2003) 5(1):59–63. doi: 10.1038/ncb898 12483219

[139] Perlson E Michaelevski I Kowalsman N Ben-Yaakov K Shaked M Seger R. Vimentin binding to phosphorylated erk sterically hinders enzymatic dephosphorylation of the kinase. J Mol Biol (2006) 364(5):938–44. doi: 10.1016/j.jmb.2006.09.056 17046786

[140] Stevens C Henderson P Nimmo ER Soares DC Dogan B Simpson KW. The intermediate filament protein, vimentin, is a regulator of NOD2 activity. Gut (2013) 62(5):695–707. doi: 10.1136/gutjnl-2011-301775 PMC4225453 22684479

[141] dos Santos G Rogel MR Baker MA Troken JR Urich D Morales-Nebreda L. Vimentin regulates activation of the NLRP3 inflammasome. Nat Commun (2015) 6:6574. doi: 10.1038/ncomms7574 PMC4358756 25762200

[142] Prahlad V Yoon M Moir RD Vale RD Goldman RD. Rapid movements of vimentin on microtubule tracks: kinesin-dependent assembly of intermediate filament networks. J Cell Biol (1998) 143(1):159–70. doi: 10.1083/jcb.143.1.159 PMC2132817 9763428

[143] Martinon F Petrilli V Mayor A Tardivel A Tschopp J. Gout-associated uric acid crystals activate the NALP3 inflammasome. Nature (2006) 440(7081):237–41. doi: 10.1038/nature04516 16407889

[144] Misawa T Takahama M Kozaki T Lee H Zou J Saitoh T. Microtubule-driven spatial arrangement of mitochondria promotes activation of the NLRP3 inflammasome. Nat Immunol (2013) 14(5):454–60. doi: 10.1038/ni.2550 23502856

[145] Liantinioti G Argyris AA Protogerou AD Vlachoyiannopoulos P. The role of colchicine in the treatment of autoinflammatory diseases. Curr Pharm Des (2018) 24(6):690–4. doi: 10.2174/1381612824666180116095658 29336247

[146] Bonaventura A Vecchie A Dagna L Tangianu F Abbate A Dentali F. Colchicine for COVID-19: targeting NLRP3 inflammasome to blunt hyperinflammation. Inflammation Res (2022) 71(3):293–307. doi: 10.1007/s00011-022-01540-y PMC8811745 35113170

[147] Yang J Zou L Yang Y Yuan J Hu Z Liu H. Superficial vimentin mediates DENV-2 infection of vascular endothelial cells. Sci Rep (2016) 6:38372. doi: 10.1038/srep38372 27910934 PMC5133558

[148] Gladue DP O'Donnell V Baker-Branstetter R Holinka LG Pacheco JM Fernandez Sainz I. Foot-and-mouth disease virus modulates cellular vimentin for virus survival. J Virol (2013) 87(12):6794–803. doi: 10.1128/JVI.00448-13 PMC3676138 23576498

[149] Koch CM Anekalla KR Hu Y-S Davis JM Ciesielski M Gadhvi G. Influenza-induced activation of recruited alveolar macrophages during the early inflammatory phase drives lung injury and lethality. bioRxiv (2022):2020.06.08.141309. doi: 10.1101/2020.06.08.141309 35923321

[150] Sabbah A Chang TH Harnack R Frohlich V Tominaga K Dube PH. Activation of innate immune antiviral responses by Nod2. Nat Immunol (2009) 10(10):1073–80. doi: 10.1038/ni.1782 PMC2752345 19701189

[151] Coulombe F Fiola S Akira S Cormier Y Gosselin J. Muramyl dipeptide induces NOD2-dependent Ly6C(high) monocyte recruitment to the lungs and protects against influenza virus infection. PloS One (2012) 7(5):e36734. doi: 10.1371/journal.pone.0036734 22590599 PMC3348889

[152] Harris J VanPatten S Deen NS Al-Abed Y Morand EF. Rediscovering MIF: New tricks for an old cytokine. Trends Immunol (2019) 40(5):447–62. doi: 10.1016/j.it.2019.03.002 30962001

[153] Al-Abed Y Dabideen D Aljabari B Valster A Messmer D Ochani M. ISO-1 binding to the tautomerase active site of MIF inhibits its pro-inflammatory activity and increases survival in severe sepsis. J Biol Chem (2005) 280(44):36541–4. doi: 10.1074/jbc.C500243200 16115897

[154] Bloom J Metz C Nalawade S Casabar J Cheng KF He M. Identification of iguratimod as an inhibitor of macrophage migration inhibitory factor (MIF) with steroid-sparing potential. J Biol Chem (2016) 291(51):26502–14. doi: 10.1074/jbc.M116.743328 PMC5159510 27793992

[155] Fingerle-Rowson G Petrenko O Metz CN Forsthuber TG Mitchell R Huss R. The p53-dependent effects of macrophage migration inhibitory factor revealed by gene targeting. Proc Natl Acad Sci U.S.A. (2003) 100(16):9354–9. doi: 10.1073/pnas.1533295100 PMC170922 12878730

[156] Bloom J Sun S Al-Abed Y. MIF, a controversial cytokine: a review of structural features, challenges, and opportunities for drug development. Expert Opin Ther Targets (2016) 20(12):1463–75. doi: 10.1080/14728222.2016.1251582 27762152

[157] Flieger O Engling A Bucala R Lue H Nickel W Bernhagen J. Regulated secretion of macrophage migration inhibitory factor is mediated by a non-classical pathway involving an ABC transporter. FEBS Lett (2003) 551(1-3):78–86. doi: 10.1016/s0014-5793(03)00900-1 12965208

[158] Merk M Baugh J Zierow S Leng L Pal U Lee SJ. The golgi-associated protein p115 mediates the secretion of macrophage migration inhibitory factor. J Immunol (2009) 182(11):6896–906. doi: 10.4049/jimmunol.0803710 PMC3135652 19454686

[159] Dankers W Hasnat MA Swann V Alharbi A Lee JP Cristofaro MA. Necrotic cell death increases the release of macrophage migration inhibitory factor by monocytes/macrophages. Immunol Cell Biol (2020) 98(9):782–90. doi: 10.1111/imcb.12376 32654231

[160] Roth S Agthe M Eickhoff S Moller S Karsten CM Borregaard N. Secondary necrotic neutrophils release interleukin-16C and macrophage migration inhibitory factor from stores in the cytosol. Cell Death Discovery (2015) 1:15056. doi: 10.1038/cddiscovery.2015.56 27551482 PMC4979515

[161] Calandra T Bernhagen J Metz CN Spiegel LA Bacher M Donnelly T. MIF as a glucocorticoid-induced modulator of cytokine production. Nature (1995) 377(6544):68–71. doi: 10.1038/377068a0 7659164

[162] Fan H Kao W Yang YH Gu R Harris J Fingerle-Rowson G. Macrophage migration inhibitory factor inhibits the antiinflammatory effects of glucocorticoids *via* glucocorticoid-induced leucine zipper. Arthritis Rheumatol (2014) 66(8):2059–70. doi: 10.1002/art.38689 PMC4116443 24782327

[163] Gunther S Fagone P Jalce G Atanasov AG Guignabert C Nicoletti F. Role of MIF and d-DT in immune-inflammatory, autoimmune, and chronic respiratory diseases: from pathogenic factors to therapeutic targets. Drug Discovery Today (2019) 24(2):428–39. doi: 10.1016/j.drudis.2018.11.003 30439447

[164] Odh G Hindemith A Rosengren AM Rosengren E Rorsman H. Isolation of a new tautomerase monitored by the conversion of d-dopachrome to 5,6-dihydroxyindole. Biochem Biophys Res Commun (1993) 197(2):619–24. doi: 10.1006/bbrc.1993.2524 8267597

[165] Vincent FB Lin E Sahhar J Ngian GS Kandane-Rathnayake R Mende R. Analysis of serum macrophage migration inhibitory factor and d-dopachrome tautomerase in systemic sclerosis. Clin Transl Immunol (2018) 7(12):e1042. doi: 10.1002/cti2.1042 PMC6283235 30546906

[166] Flores M Saavedra R Bautista R Viedma R Tenorio EP Leng L. Macrophage migration inhibitory factor (MIF) is critical for the host resistance against toxoplasma gondii. FASEB J (2008) 22(10):3661–71. doi: 10.1096/fj.08-111666 PMC2537436 18606868

[167] Stojanovic I Mirkov I Kataranovski M Glamoclija J Stosic-Grujicic S. A role for macrophage migration inhibitory factor in protective immunity against aspergillus fumigatus. Immunobiology (2011) 216(9):1018–27. doi: 10.1016/j.imbio.2011.03.005 21489649

[168] Galvao I Dias AC Tavares LD Rodrigues IP Queiroz-Junior CM Costa VV. Macrophage migration inhibitory factor drives neutrophil accumulation by facilitating IL-1beta production in a murine model of acute gout. J Leukoc Biol (2016) 99(6):1035–43. doi: 10.1189/jlb.3MA0915-418R PMC4952013 26868525

[169] Shin MS Kang Y Wahl ER Park HJ Lazova R Leng L. Macrophage migration inhibitory factor regulates U1 small nuclear RNP immune complex-mediated activation of the NLRP3 inflammasome. Arthritis Rheumatol (2019) 71(1):109–20. doi: 10.1002/art.40672 PMC6310104 30009530

[170] Kim MJ Kim WS Kim DO Byun JE Huy H Lee SY. Macrophage migration inhibitory factor interacts with thioredoxin-interacting protein and induces NF-kappaB activity. Cell Signal (2017) 34:110–20. doi: 10.1016/j.cellsig.2017.03.007 28323005

[171] Koebernick H Grode L David JR Rohde W Rolph MS Mittrucker HW. Macrophage migration inhibitory factor (MIF) plays a pivotal role in immunity against salmonella typhimurium. Proc Natl Acad Sci U.S.A. (2002) 99(21):13681–6. doi: 10.1073/pnas.212488699 PMC129741 12271144

[172] Reyes JL Terrazas LI Espinoza B Cruz-Robles D Soto V Rivera-Montoya I. Macrophage migration inhibitory factor contributes to host defense against acute trypanosoma cruzi infection. Infect Immun (2006) 74(6):3170–9. doi: 10.1128/IAI.01648-05 PMC1479264 16714544

[173] Roger T Delaloye J Chanson AL Giddey M Le Roy D Calandra T. Macrophage migration inhibitory factor deficiency is associated with impaired killing of gram-negative bacteria by macrophages and increased susceptibility to klebsiella pneumoniae sepsis. J Infect Dis (2013) 207(2):331–9. doi: 10.1093/infdis/jis673 23125447

[174] Herrero LJ Nelson M Srikiatkhachorn A Gu R Anantapreecha S Fingerle-Rowson G. Critical role for macrophage migration inhibitory factor (MIF) in Ross river virus-induced arthritis and myositis. Proc Natl Acad Sci U.S.A. (2011) 108(29):12048–53. doi: 10.1073/pnas.1101089108 PMC3141998 21730129

[175] Lai YC Chuang YC Chang CP Lin YS Perng GC Wu HC. Minocycline suppresses dengue virus replication by down-regulation of macrophage migration inhibitory factor-induced autophagy. Antiviral Res (2018) 155:28–38. doi: 10.1016/j.antiviral.2018.05.002 29752950

[176] Guo J Huang F Liu J Chen Y Wang W Cao B. The serum profile of hypercytokinemia factors identified in H7N9-infected patients can predict fatal outcomes. Sci Rep (2015) 5:10942. doi: 10.1038/srep10942 26028236 PMC4450576

[177] Yang Y Wong G Yang L Tan S Li J Bai B. Comparison between human infections caused by highly and low pathogenic H7N9 avian influenza viruses in wave five: Clinical and virological findings. J Infect (2019) 78(3):241–8. doi: 10.1016/j.jinf.2019.01.005 30664912

[178] Hou XQ Gao YW Yang ST Wang CY Ma ZY Xia XZ. Role of macrophage migration inhibitory factor in influenza H5N1 virus pneumonia. Acta Virol (2009) 53(4):225–31. doi: 10.4149/av_2009_04_225 19941385

[179] Rodrigues TS de Sa KSG Ishimoto AY Becerra A Oliveira S Almeida L. Inflammasomes are activated in response to SARS-CoV-2 infection and are associated with COVID-19 severity in patients. J Exp Med (2021) 218(3):e20201707. doi: 10.1084/jem.20201707 33231615 PMC7684031

[180] Zeng J Xie X Feng XL Xu L Han JB Yu D. Specific inhibition of the NLRP3 inflammasome suppresses immune overactivation and alleviates COVID-19 like pathology in mice. EBioMedicine (2022) 75:103803. doi: 10.1016/j.ebiom.2021.103803 34979342 PMC8719059

[181] Coates BM Staricha KL Ravindran N Koch CM Cheng Y Davis JM. Inhibition of the NOD-like receptor protein 3 inflammasome is protective in juvenile influenza a virus infection. Front Immunol (2017) 8:782. doi: 10.3389/fimmu.2017.00782 28740490 PMC5502347

[182] Tate MD Ong JDH Dowling JK McAuley JL Robertson AB Latz E. Reassessing the role of the NLRP3 inflammasome during pathogenic influenza a virus infection *via* temporal inhibition. Sci Rep (2016) 6:27912. doi: 10.1038/srep27912 27283237 PMC4901306

[183] Guo C Fu R Wang S Huang Y Li X Zhou M. NLRP3 inflammasome activation contributes to the pathogenesis of rheumatoid arthritis. Clin Exp Immunol (2018) 194(2):231–43. doi: 10.1111/cei.13167 PMC6194337 30277570

[184] Mullard A. NLRP3 inhibitors stoke anti-inflammatory ambitions. Nat Rev Drug Discovery (2019) 18(6):405–7. doi: 10.1038/d41573-019-00086-9 31160775

[185] Park A Iwasaki A. Type I and type III interferons - induction, signaling, evasion, and application to combat COVID-19. Cell Host Microbe (2020) 27(6):870–8. doi: 10.1016/j.chom.2020.05.008 PMC7255347 32464097

[186] Reyes LF Rodriguez A Bastidas A Parra-Tanoux D Fuentes YV Garcia-Gallo E. Dexamethasone as risk-factor for ICU-acquired respiratory tract infections in severe COVID-19. J Crit Care (2022) 69:154014. doi: 10.1016/j.jcrc.2022.154014 35217370 PMC8863516

[187] Rothe K Lahmer T Rasch S Schneider J Spinner CD Wallnofer F. Dexamethasone therapy and rates of secondary pulmonary and bloodstream infections in critically ill COVID-19 patients. Multidiscip Respir Med (2021) 16(1):793. doi: 10.4081/mrm.2021.793 34760275 PMC8567088

[188] Xing X Hu S Chen M Zhan F Liu H Chen Z. Severe acute respiratory infection risk following glucocorticosteroid treatment in uncomplicated influenza-like illness resulting from pH1N1 influenza infection: a case control study. BMC Infect Dis (2019) 19(1):1080. doi: 10.1186/s12879-019-4669-9 PMC6933691 31878888

[189] Larsson H Ahren B. Insulin resistant subjects lack islet adaptation to short-term dexamethasone-induced reduction in insulin sensitivity. Diabetologia (1999) 42(8):936–43. doi: 10.1007/s001250051251 10491753

[190] Guo X Chen S Yu W Chi Z Wang Z Xu T. AKT controls NLRP3 inflammasome activation by inducing DDX3X phosphorylation. FEBS Lett (2021) 595(19):2447–62. doi: 10.1002/1873-3468.14175 34387860

[191] Cho Y Crichlow GV Vermeire JJ Leng L Du X Hodsdon ME. Allosteric inhibition of macrophage migration inhibitory factor revealed by ibudilast. Proc Natl Acad Sci U.S.A. (2010) 107(25):11313–8. doi: 10.1073/pnas.1002716107 PMC2895110 20534506

[192] Martinez-Guerra BA Gonzalez-Lara MF Roman-Montes CM Tamez-Torres KM Dardon-Fierro FE Rajme-Lopez S. Outcomes of patients with severe and critical COVID-19 treated with dexamethasone: a prospective cohort study. Emerg Microbes Infect (2022) 11(1):50–9. doi: 10.1080/22221751.2021.2011619 PMC8725849 34839785

[193] Tang C Wang Y Lv H Guan Z Gu J. Caution against corticosteroid-based COVID-19 treatment. Lancet (2020) 395(10239):1759–60. doi: 10.1016/S0140-6736(20)30749-2 PMC7247780 32464115

